# Modeled Groundwater Decline is Associated with Lower Arsenic Concentrations in Hanoi

**DOI:** 10.1021/acs.est.6c07700

**Published:** 2026-10-18

**Authors:** David Haaf, Michael McGlade, Athena A. Nghiem, Duyen Thi Vu, Tobias Steinert, Viet Hung Pham, Linh Khanh Bui, Thanh Ngoc Tong, Duong Du Bui, Andrea Stenke, Michael Berg, Lenny H. E. Winkel

**Affiliations:** † Department of Water Resources and Drinking Water, Eawag: Swiss Federal Institute of Aquatic Science and Technology, Dubendorf 8600, Switzerland; ‡ Department of Environmental Systems Science (D-USYS), Institute of Biogeochemistry and Pollutant Dynamics, ETH Zurich, Zurich 8092, Switzerland; § Department of Geoscience, University of Wisconsin–Madison, Madison, Wisconsin 53706, United States; ∥ Key Laboratory of Analytical Technology for Environmental Quality and Food Safety Control (KLATEFOS), VNU University of Science, Hanoi 11417, Vietnam; ⊥ National Center for Water Resources Planning and Investigation (NAWAPI), Water Research Institute, Hanoi 10000, Vietnam; # Vietnam Water and Environment Association, Hanoi 11307, Vietnam

**Keywords:** hydrochemical dynamics, redox processes, decadal change, groundwater abstraction, aquifer flushing, cone of depression, CatBoost modeling, SHAP

## Abstract

Elevated arsenic concentrations in groundwater pose health risks. Geogenic arsenic contamination is driven by biogeochemical mobilization and groundwater dynamics. Intensive pumping can initially exacerbate contamination by drawing arsenic or mobilizing organic matter into aquifers, but over longer time scales may also promote dilution and flushing in some settings. To better understand the mechanisms controlling arsenic concentrations, we examined changes over two decades in Hanoi (Vietnam), a hotspot of geogenic arsenic contamination and groundwater pumping. Arsenic concentrations from 470 wells sampled in the year 2000 were compared with 434 wells sampled in the same area in 2013 and 2023. Median concentrations declined from 14 to 8 μg/L between 2000 and 2023, while mean concentrations and the proportion of high-arsenic wells (>100 μg/L) remained similar. Using hydrochemical and groundwater-level data, CatBoost models combined with SHAP analysis assessed changes in predictor importance. In 2000, arsenic was more strongly associated with redox predictors, whereas groundwater-level predictors became more important in 2023. Within wells used to evaluate groundwater-level change (*n* = 251), larger decadal declines were associated with lower arsenic concentrations (median = 31 μg/L) than wells without declines (median = 120 μg/L). We hypothesize a decadal mechanism shift, from redox to hydrological controls, particularly driving arsenic declines.

## Graphical abstract

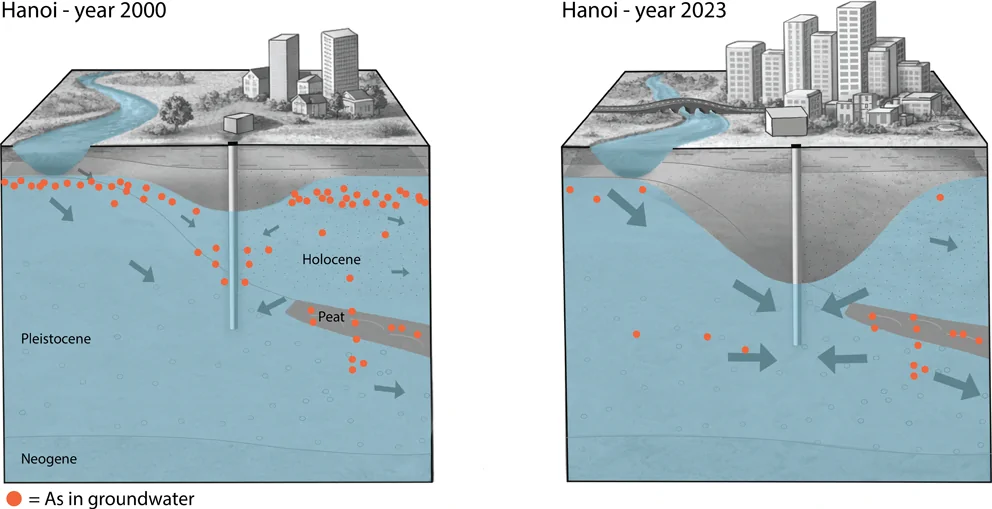

## 1 Introduction

Groundwater contamination with the toxic and carcinogenic element arsenic (As) is a critical global public-health priority.^1–3^ While the World Health Organization (WHO) recommends a maximum arsenic (As) concentration of 10 μg/L in drinking water, levels in groundwater in the river deltas of South-East Asia frequently exceed this threshold.^4–7^ These elevated concentrations are primarily attributed to natural biogeochemical processes, particularly the reductive dissolution of As-bearing iron (oxyhydr)­oxides, in which dissolved organic matter (DOM) acts as an electron donor.^1,3,8,9^

However, As contamination is also influenced by groundwater dynamics, including groundwater levels, flow direction, and velocity. Over the past century, anthropogenic groundwater abstraction has increased, particularly in highly urbanized regions of South and South-East Asia that are recognized hotspots of geogenic As contamination.^10^ This persistent abstraction has formed large groundwater cones of depression in cities such as Dhaka, Bangladesh,^11,12^ Chiang Mai, Thailand,^13^ and Hanoi, Vietnam.^14,15^ These cones can modify hydraulic gradients, redistributing groundwater As by drawing contaminated groundwater or As-mobilizing compounds from surrounding aquifers and clay-rich sediments into deeper aquifers.^16–20^ Groundwater drawdown can also promote lateral advection of As from riverbed and bank sediments into typically low-As aquifers.^21–25^ Over longer time scales, such altered hydraulic gradients may additionally modify recharge pathways and groundwater replacement processes, potentially contributing to flushing and dilution in some hydrogeological settings.^26^

Although these mechanisms are well established, most evidence derives from short-term or seasonal observations from limited numbers of wells in spatially restricted areas, leaving uncertainty about how sustained pumping alters As distributions over longer time scales. For example, in Titas district, Bangladesh, a year-long study resampling six wells concluded that dry-season groundwater drawdown compressed clay layers, releasing DOM and increasing As concentrations, with a delayed response during wet-season relaxation.^27^ Regional groundwater flow changes can also redistribute As-rich water between aquifers. In particular, variations in pumping and seasonal recharge have been shown in some settings to facilitate the potential movement of As-rich groundwater from shallow Holocene aquifers into deeper Pleistocene layers.^17,28–30^ These processes involve reversible As desorption and adsorption along groundwater flow paths over seasonal to annual time scales.^19,29,31,32^

Conversely, studies in Hanoi and other deltas suggest that these modified hydraulic gradients, often driven by long-term pumping, can have the opposite effect: promoting partial flushing and removal of As from affected aquifers.^22,30,33–36^ Postma et al.^22^ showed that at the Nam Du site near Hanoi, pumping-induced infiltration of Red River water led to groundwater replacement and declining As concentrations, based on observations from approximately 15 monitoring wells; however, decreases were gradual due to strong sediment adsorption. Hoang et al.^26^ suggested that sustained pumping across the Red River Delta has altered hydraulic gradients through expanding cones of depression, potentially inducing riverbank infiltration, groundwater replacement, and aquifer flushing in some hydrogeological settings. Although these studies provide mechanistic insights into As flushing, they are often limited in spatial extent, duration, and sample size. It remains unclear whether long-term, large-scale pumping increases As concentrations through redistribution or decreases it through flushing, particularly over decadal time scales and large spatial areas. Addressing this uncertainty requires approaches capable of analyzing large, spatially distributed data sets and resolving complex, nonlinear hydrochemical and hydrological interactions that are difficult to capture using statistical models.

Machine learning offers a promising approach to identify the drivers of As contamination (e.g., redox controls and groundwater level changes) and to detect potential changes in concentration over time.^2,37,38^ Models such as Random Forest, CatBoost, and Neural Networks, combined with SHapley Additive exPlanations (SHAP), can rank variable importance, and identify dominant controls on As concentrations.^39–41^

This study assesses whether sustained pumping across hundreds of wells over two decades across a large spatial extent (∼970 km^2^) alters groundwater As concentrations. By integrating hydrochemical observations with groundwater level dynamics in a machine learning framework, we identify how sustained pumping is associated with changes in the relative importance of predictors controlling As. Specifically, we use multiple machine learning approaches coupled with SHAP and double/debiased machine learning to capture nonlinear hydrochemical and hydrological processes and characterize their associations on As contamination over decadal time scales. This approach aims to help address the long-standing uncertainty over whether sustained pumping generally increases As concentrations through mechanisms such as redox-driven mobilization, clay layer compaction, and pumping-induced groundwater drawdown, or instead may lower As through flushing. We analyze multidecadal changes in groundwater As in the Greater Hanoi region by comparing groundwater samples collected in 2023 with data from the same area surveyed in 2013/14 ^46^ and 2000 ^42^ and use machine learning to identify the drivers of observed trends.

## 2 Methods

### 2.1 Study Area and Groundwater Sampling Campaigns

All analyses of groundwater As changes were carried out in Greater Hanoi (20.9 °N−21.5 °N; 105.8 °E−106.3 °E), which covers approximately 970 km^2^ (Figure 1). This region has undergone rapid urban expansion in recent decades; for example, the urban area reached approximately 565 km^2^ in 2020, of which ∼34% developed between 2010 and 2020.^43^ This expansion has been accompanied by extensive groundwater pumping and widespread As levels exceeding WHO guidelines.^20,24,44–46^ It should be noted that the Hanoi drinking water utilities treat groundwater to remove As before the water is piped to supply the city.^44^

**Figure 1:**
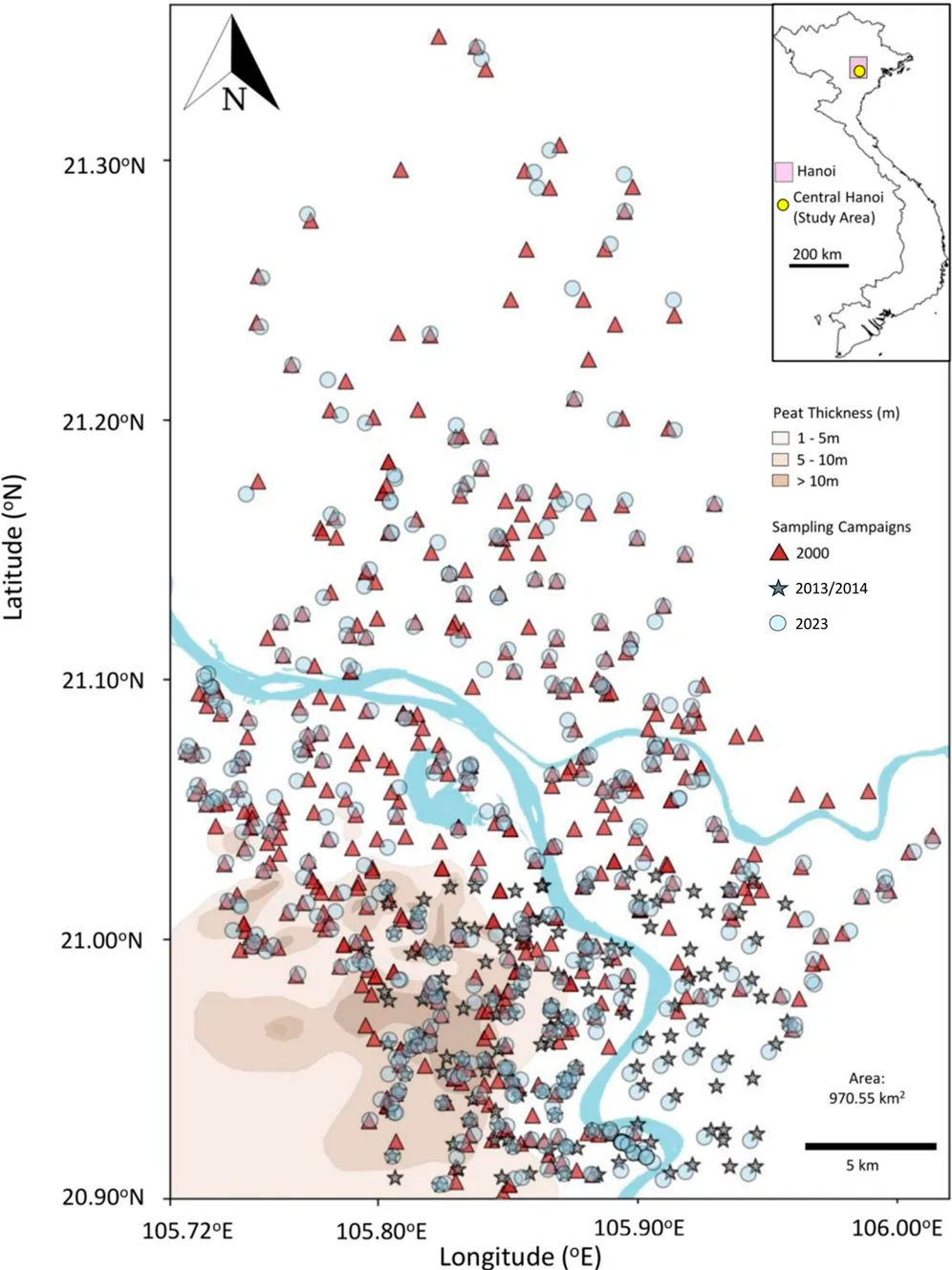
Hanoi region (pink box, top right) highlighting the study area in central Hanoi (yellow circle; approximately 970 km^2^). Groundwater wells sampled in 2000 (red triangles; Tong et al., 2001), 2013/14 (gray stars; Nghiem et al., 2019), and 2023 (blue circles; this study) are shown. A positional offset (500 m to the northeast) was applied to distinguish overlapping sampling locations. Brown shaded contours indicate the presence of peat/organic-rich sediments and their estimated thicknesses: 1−5 m (light), 5−10 m (medium), and >10 m (dark) (Trafford et al., 1996) for the purposes of showing potential dissolved organic carbon sources.

We use existing As data from a groundwater sampling campaign conducted by the Northern Hydrogeological and Engineering Geological Division (NHEGD) in November 2000 ^42^ (Figure 1). In this campaign, 470 groundwater wells (private household, public, and monitoring) were sampled across the Greater Hanoi area (20.90–21.35° N; 105.72–106.01° E), referred to here as the “2000 data set.” In addition, data from a more recent campaign targeting private household wells were collected in a smaller subregion of the study area between May and December 2013 and in March 2014 ^46^ (Figure 1). This campaign analyzed 137 wells in southern Hanoi (20.91–21.17° N; 105.80–105.96° E) and is referred to as the “2013/14 data set.” To assess changes in As distribution over two decades, we conducted a sampling campaign between February and June 2023, analyzing 434 wells across the Greater Hanoi region (Figure 1), hereafter referred to as the “2023 dataset” (Supporting Information).

The 2023 campaign prioritized resampling wells from previous campaigns (2000 and 2013/14) where possible. When a well sampled in 2000 no longer existed or could not be confidently reidentified, the nearest available well was selected as a replacement to retain approximately comparable spatial coverage. In contrast, the status of wells sampled in 2013/14 was known in advance, facilitating their relocation. As a result, each data set was treated as independent, with analyses focused on general spatiotemporal patterns of As contamination rather than well-specific trends. The analysis reflects large-scale spatial changes rather than well-specific temporal changes, and causal relationships between changes in As and hydrological or hydrochemical variables cannot be directly established.

The 2023 data set (*n* = 434) is used in its entirety for comparison with the 2000 data set (*n* = 470) to assess temporal changes in As across the Greater Hanoi area. The 2013/14 data set (*n* = 137) is used only in a separate, spatially restricted subanalysis to illustrate more granular temporal shifts in As in southern Hanoi, using subsets of the 2000 (*n* = 146) and 2023 (*n* = 141) well data, spatially present in the subregion of the 2013/14 data set. Importantly, As concentrations could not be compared across detailed well depths because such information was not provided in the 2000 NHEGD data set; only aquifer assignment (Holocene or Pleistocene), as reported by NHEGD, was available.

### 2.2 Groundwater Sampling

Groundwater sampling followed established protocols conducted for the Red River Delta.^24,44,47^ Wells were pumped until field parameters stabilized and were measured using a portable meter Hach HQ40d, including pH (PHC201), Eh (MTC301), dissolved oxygen (LDO101), electrical conductivity (CDC401), and built-in temperature sensor. Trace element and major cations were determined by inductively coupled plasma mass spectrometry (ICP–MS; Agilent 8900 Triple Quadrupole). Major anions were measured by ion chromatography (Metrohm 930 Compact IC Flex). At least three quality-control checks were performed within each of the four analytical batches. Calibration curves showed *R* ^2^ ≥ 0.998, and recoveries exceeded 95% for all measured elements across all batches. Concentrations below the analyte-specific LOQ were assigned the corresponding LOQ value. Field and laboratory blanks showed no anomalies. Full sampling, analytical and quality-control procedures are provided in the Supporting Information (Methods Sections 2.3; Figures SI1–SI2; Tables SI1–SI4).^48,50^

### 2.3 Data Quality Check

We assessed the reliability of the 2000, 2013/14, and 2023 data sets by comparing field parameters and ICP–MS results with benchmark data from Winkel et al.^20^ and Nghiem et al.^46^ (Table 1). We examined data distributions for systematic offsets, outliers, and range disparities (Tables S3 and S4, Supporting Information). Field parameters from the 2000 campaign (pH, Eh, EC) were excluded from further analyses due to inconsistencies with benchmarks (Supporting Information 1).

**Table 1:** Sampling Campaigns and Benchmark Datasets Used in This Study^a^

campaign	study	no. well	sampling period	measurement parameters	LOQ (As) (μg/L)
2000	Tong et al.^42^	470	Nov. 2000	Eh, pH, EC, DO, HCO_3_^−^, As, Fe, Mn, NH_4_^+^	1
2013/14	Nghiem et al.^46^	137	May−Dec. 2013; Mar. 2014	Eh, pH, EC, As, Fe, Mn, Cl^−^, Br^−^, F^−^, SO_4_^2−^, P, K, Na, Ca, Mg, DOC	0.2
2023	This study	434	Feb.−June, 2023	Eh, pH, EC, DO, HCO_3_^−^, As, Fe, Mn, NH_4_^+^, Cl^−^, Br^−^, F^−^, SO_4_^2−^, NO_3_^−^−N, P, S, K, Sr, Mo, Na, Si, Ca, Mg, Ba, DOC, CH_4_	0.8
2005−07	Winkel et al.^20^	512	May 2005−Jan. 2007	Eh, pH, EC, DO, HCO_3_^−^, As, Fe, Mn, NH_4_^+^, Cl^−^, Br^−^, SO_4_^2−^, NO_3_^−^−N, P, S, K, Sr, Mo, Na, Si, Ca, Mg, Ba, DOC	1

a Reported sample size, sampling period, hydrochemical parameters, and As LOQ are provided. The 2000 and 2023 campaigns form the primary datasets used for the decadal comparison. The 2013/14 dataset (Nghiem et al., 2019) provides an intermediate benchmark and is used for a spatially restricted sub-analysis in southern Hanoi.

### 2.4 Estimating Groundwater Level Changes

We used a regional MODFLOW model of the Red River Delta to simulate monthly groundwater-level (GWL) data from 2000 to 2020. The model was parametrized using regional hydrogeological data (e.g., recharge estimates, GWL heads, aquifer information, and hydraulic conductivity) and calibrated against GWL measurements from 120 monitoring wells.^14^ The GWL was simulated across a 250 × 250 m grid covering the delta region (20−22°N, 105−107°E). Detailed procedure of MODFLOW simulations is provided in Supporting Information 2. Modeled GWL (hydraulic head) was extracted at well locations using available well coordinate and aquifer classification information. Because groundwater sampling occurred in 2023, but MODFLOW simulations only extend until 2020, we evaluated annual GWL trends over the simulation period to assess whether 2020 GWL is representative of conditions in 2023. A Mann−Kendall trend test was used to evaluate annual GWL changes. This test was selected because it is well suited to time-series data, does not assume normal data distribution, and is robust to outliers. Groundwater level declined significantly between 2000 and 2015 (τ = −0.78, *p* < 0.0001; Figure SI3, Supporting Information), but showed no significant change thereafter (2015−2020; τ = − 0.09, *p* = 0.329), indicating a GWL stabilization. This stabilization supports the assumption that 2020 GWL reasonably represents 2023 conditions, although this could not be verified because simulations were unavailable for 2021−2023.

For all wells sampled during the 2023 campaign, GWL change relative to 2000 was estimated as follows. (i) The coordinates of all 434 wells were assigned to the corresponding 250 × 250 m MODFLOW grid cell in which each well is located. (ii) Monthly GWL values for 2000 and 2020 were extracted at each well location. (iii) Seasonal mean GWL was then calculated for the years 2000 and 2020, for both the wet season (May−October) and the dry season (January−April plus November−December). These seasons were defined using daily rainfall data for the Greater Hanoi region derived from the European Centre for Medium-Range Weather Forecasts.^49^ Furthermore, we assessed whether antecedent rainfall could influence GWL during 2000, 2013/14, and 2020, and found no (strong) indications of exceptionally wet or dry antecedent conditions (Figure SI5, Supporting Information). (iv) Seasonal GWL changes were computed by comparing 2000 and 2023 means within the same season. *Z*-score analysis of simulated GWL values across the 2000−2020 simulation period indicated that GWL in 2000 fell within the normal range of modeled conditions for both aquifers (Holocene: *z* = 0.91, *p* = 0.36; Pleistocene: *z* = 1.01, *p* = 0.31). (v) Statistical differences between seasonal GWL values in 2000 and 2020 were assessed using a Wilcoxon signed-rank test for each well individually. This test was selected because the analysis involves paired measurements and does not assume normally distributed differences between observations. Wells were classified as showing either a significant (*p* < 0.05) or nonsignificant (*p* ≥ 0.05) change in GWL. (vi) Wells were grouped into four categories: dry season/no significant GWL change, dry season/significant GWL change, wet season/no significant GWL change, and wet season/significant GWL change (Figure SI6). (vii) As concentrations were compared between these groups.

### 2.5 Machine Learning and Variable Importance

#### 2.5.1 Model Selection

Machine learning was used to assess whether the relative importance of predictors associated with As contamination differed across decades, specifically whether there was a relative change in the importance of redox-related and hydrochemical predictors potentially influenced by pumping. This analysis was conducted separately for the 2000 and 2023 campaigns. Separate CatBoost models were trained for each data set, with SHAP (SHapley Additive exPlanations) used to quantify changes in relative predictor importance. CatBoost was selected for its strong performance on small data sets^51^ and its flexibility to handle regression and classification problems.^52^ For example, Ibrahim et al.^37^ demonstrated strong predictive performance of CatBoost models trained on hydrochemical variables to predict As mobility using a data set size (*n* = 597) comparable to our sampling campaigns. Moreover, CatBoost outperformed other modeling evaluated in this study, including random forests and linear regression (see Supporting Information 3).

#### 2.5.2 Model Development/Optimization

Several predictors, including pH, Eh and EC, were excluded because the corresponding field measurements from the 2000 campaign were inconsistent with benchmark data sets (Supporting Information 1). Other relevant variables, such as DOC, were unavailable for 2000 and therefore could not be included consistently across both data sets (Table 1). Variables available in both campaigns, including alkalinity and dissolved oxygen, were assessed using permutation importance on the held-out test data set. Each predictor was randomly shuffled, and the change in test *R* ^2^ quantified its predictive contribution. Performance decreases for alkalinity (Δ*R* ^2^ ≈ 0.06) and dissolved oxygen (Δ*R* ^2^ ≈ 0.01) indicated limited additional predictive value, supporting exclusion. This does not imply these variables are hydrochemically unimportant, but that they provided limited predictive information in these models. Numerical well depth data were available for 2023 but not for 2000, for which only aquifer classifications were supplied. Depth was therefore excluded because it could not be incorporated consistently across both campaign-specific models.

Aquifer classification was excluded because it is a broad binary descriptor that does not capture numerical depth or within-aquifer heterogeneity. Furthermore, it was also used to assign aquifer-specific GWL estimates, so aquifer classification and GWL contained partly overlapping information. Including both could redistribute SHAP contributions and complicate interpretation. Aquifer contribution was therefore evaluated through sensitivity analysis (Section 3.4).

Based on the variable exclusion screening above, our modeling included four predictor variables: Fe, NH_4_^+^, Mn, and GWL change; however, these predictors do not represent the full range of hydrochemical, geological and hydrological factors that may influence groundwater As concentrations. However, given these constraints, the feature set used for the modeling analysis was of good quality and represented the best available set of consistently measured variables across both campaigns. Moreover, NH_4_^+^, Mn and Fe are established redox-related parameters relevant to groundwater As mobilization.

The training data set size comprised of 461 wells for 2000 and 344 wells for 2023. The CatBoost regression models were implemented in Python (v3.13.7) with As set as the target and NH_4_^+^, Mn, Fe, and seasonal mean GWL as predictors. Arsenic concentrations were modeled on the untransformed scale (μg L^−1^). These variables were selected for several reasons. Ammonium is a common redox proxy, with elevated levels indicating reducing conditions.^53^ Elevated NH_4_^+^ may reflect peat-derived organic nitrogen mineralization, supplying labile DOC that promotes Fe-oxide reduction and As mobilization.^21^ Manganese reduction can release sorbed As, making Mn a potential contributor to As mobilization.^54^ Iron is closely linked to As geochemistry, as reductive dissolution of Fe (oxyhydr)­oxides releases sorbed As. Groundwater level was included because pumping-induced drawdown may alter hydraulic gradients and recharge pathways, potentially influencing groundwater replacement and flushing processes.^22^

Data adequacy was evaluated using the sample-to-feature ratio (SFR) following Zhu et al.^55^ who identify SFR ≥10 as the minimum recommended threshold for supervised learning. In this study, SFR values were 115 (2000 data set) and 86 (2023 data set), both considerably exceeding this minimal threshold. Using 5-fold cross-validation, the optimized training-testing splits were 85:15 (2000) and 90:10 (2023). Hyperparameters were selected using Bayesian optimization. Ordered boosting was used to minimize data leakages.^52^ Overfitting was minimized using early stopping and monitored using learning curves. For hyperparameters and learning curves, see Supporting Information 3. CatBoost reliability was evaluated using an audit analysis, with both models showing strong generalization, minimal overfitting, and high robustness and stability (Supporting Information 3, Section 3).

To account for spatial autocorrelation from clustered wells, model performance was evaluated using 5-fold spatial block cross-validation. Wells were grouped into 3 km blocks using geographical coordinates, with all wells in each block assigned to the same fold. The 3 km size grouped neighboring wells while retaining sufficient blocks for balanced cross-validation. Moran’s *I* showed that spatial autocorrelation was no longer significant beyond 3 km in either campaign (Figure SI7, Supporting Information). CatBoost performance and predictor rankings were compared with random cross-validation.

#### 2.5.3 SHAP Variable Importance Assessment

The importance of individual predictors was examined using SHAP (SHapley Additive exPlanations) values^56^ computed with the *shap* package in Python. SHAP scores quantify the contribution of each predictor to the model prediction of As concentrations. Positive SHAP scores indicate that a predictor increases the predicted As concentration, whereas negative values indicate a decrease. Larger absolute SHAP scores indicate a stronger influence of the predictor on the model output. Because SHAP rankings depend on the predictors included in each model, they were interpreted only relative to NH_4_^+^, Fe, Mn and GWL. Accordingly, a high ranking does not establish greater importance than other relevant excluded predictors, e.g., pH or Eh. Because the CatBoost models were trained independently with different predictor distributions, target distributions and predictor correlations, absolute SHAP values were not directly compared between the two data sets. Moreover, because the data sets differed in wells sampled, aquifer representation and spatial distribution, contrasts in model behavior and predictor rankings may partly reflect these data set differences rather than temporal change. These contrasts therefore cannot be attributed solely to changes in the processes controlling As concentrations. SHAP values were therefore interpreted within each model, with comparisons limited to within-model predictor rankings. These differences were interpreted as model-inferred differences in relative predictor importance rather than evidence of changing causal mechanisms.

To assess associations between individual predictors and As concentrations, we applied double/debiased machine learning (DML) using CatBoost models.^57^ DML is a framework for inference about a target parameter in the presence of nuisance functions. DML uses machine learning (i.e., CatBoost models) to estimate these nuisance functions and combines this with cross-fitting and orthogonalization to reduce bias in the estimated target parameter.^57^ Full implementation details are provided in the Supporting Information (Methods: Section 2.5). SHAP rankings and DML estimates were interpreted as model-specific predictor importance and associations, respectively, rather than as causal or mechanistic evidence. These results were instead used to evaluate whether the observed patterns were consistent with the hypothesized evolution of groundwater As contamination under changing hydrological conditions.^58^

### 2.6 Statistical Tests

To assess associations between As and different predictors, Pearson correlation analysis was applied when predictor variables were normally distributed; otherwise, Spearman rank correlation was used. Data normality was assessed using the Shapiro–Wilk test. To evaluate differences in As concentrations between groups, the Kruskal–Wallis test was applied. For significant Kruskal–Wallis results, effect size (η^2^) was calculated to quantify the proportion of variance explained, with thresholds of small (0.01 − < 0.06), medium (0.06 − < 0.14), and large (≥0.14) effects.^59^ When more than two groups were compared, pairwise differences were assessed using the Conover−Iman posthoc test.^60^ To evaluate whether the association between GWL change (Δ2020−2000) and As concentrations varied across hydrochemical conditions, we applied linear regression. Arsenic concentrations were specified as the response variable, with ΔGWL as the predictor. Methane, Fe, Mn, NH_4_^+^, Na, DOC, and SO_4_^2−^ were included as covariates to examine whether hydrochemical conditions modified the GWL−As relationship. This regression model was specified as As = β_0_ + β_1_ΔGWL + β_2_X + β_3_(ΔGWL × X) + ε, where ΔGWL is the mean seasonal GWL change and X represents each hydrochemical variable (CH_4_, Fe, Mn, NH_4_^+^, Na, DOC, and SO_4_^2−^). The “β” coefficients indicate the estimated effect of each predictor on As concentrations, while “ε” represents the regression model error.

Nearby wells in the study area may not be statistically independent, potentially introducing spatial autocorrelation. We assessed this using 3 km spatial blocks, justified in Section 2.5.2. For As comparisons between wells with and without significant GWL declines and the CH_4_ interaction analysis, we used cluster-robust regression with standard errors clustered by 3 km blocks. For the GWL−As regression and temporal As comparisons between campaigns, 3 km blocks were resampled 10,000 times to obtain 95% confidence intervals.

## 3 Results and Discussion

### 3.1 Changes in As Contamination Patterns in Greater Hanoi (2000 Compared to 2023)

The spatial distribution of groundwater As across the 470 wells sampled in 2000 and the 434 wells sampled in 2023 is shown in Figure 2a,b for Hanoi. At both time points, As concentrations were generally higher southwest (>50 μg/L), particularly around 21.0°N, 105.8°E, consistent with other field studies.^24,44–46,61^ In contrast, As concentrations were lower at both time points (<5 μg/L) in the northern parts of the study region (∼21.2 °N, 105.9 °E).

**Figure 2:**
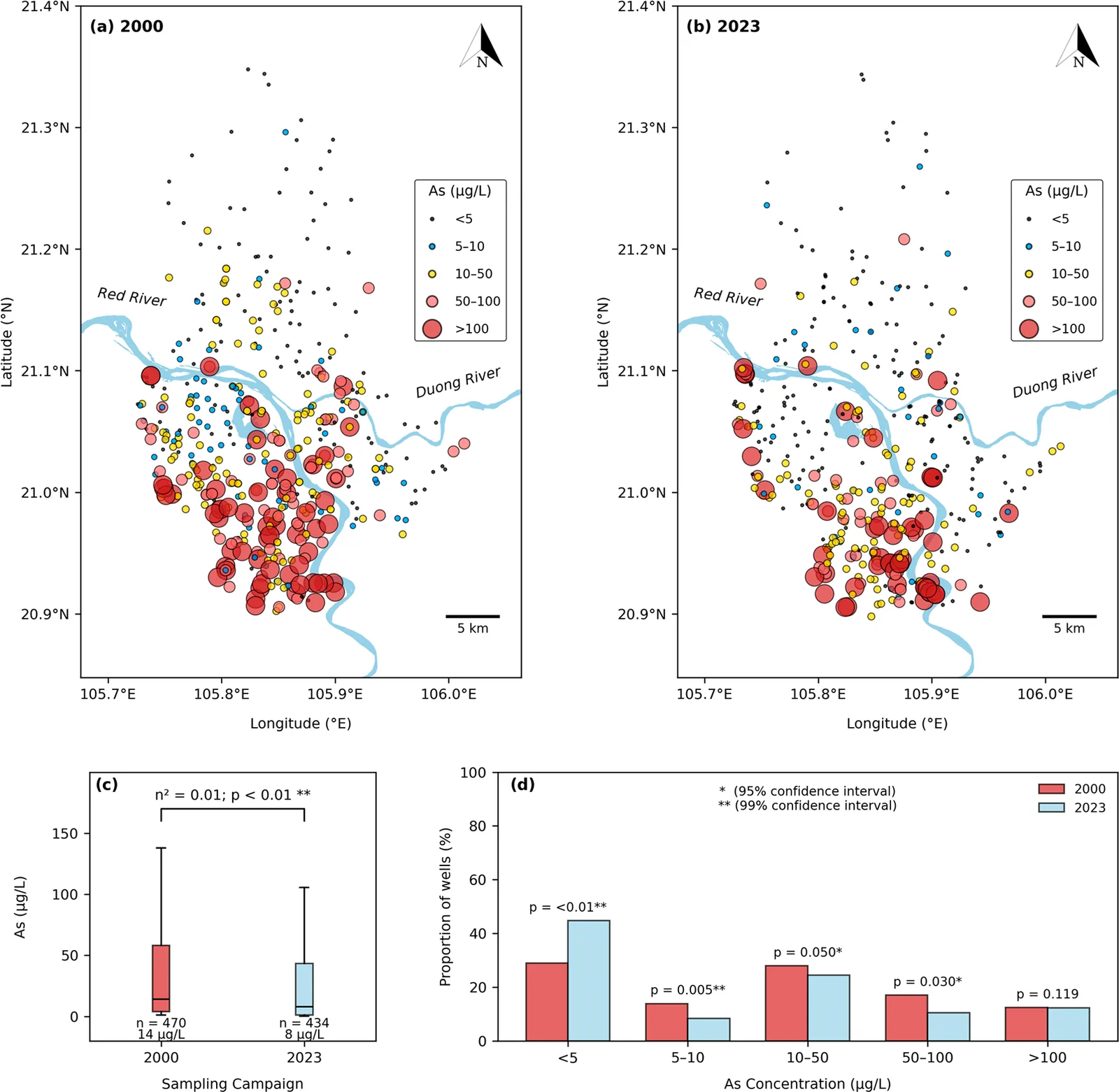
(a) As distribution in (a) 2000 (*n* = 470) and (b) 2023 (*n* = 434). Point color and size represent As concentration ranges: smaller black/blue points indicate <10 μg/L, while larger yellow/pink/red points indicate >10 μg/L. (c) Median As concentrations in 2000 (red) and 2023 (blue), with effect size (*n* ^2^) and *p*-value from the Kruskal–Wallis test. (d) Number of wells across As concentrations (<5, 5−10, 10−50, 50 −100, >100 μg/L) in 2000 (red) and 2023 (blue), with p-values for group comparisons. Asterisks indicate a statistically significant difference.

Across the two decades, As concentrations were generally higher in 2000 (median = 14 μg/L) than in 2023 (median = 8 μg/L; Figure 2c), with this difference being significant (*p* < 0.01). This remained supported after accounting for spatial clustering with a 3 km spatial-block bootstrap; the median difference (2023 − 2000) was −6.2 μg/L (95% CI: −10.4 to −1.0 μg/L). However, mean As concentrations were similar (40 μg/L in 2000; 41 μg/L in 2023), reflecting strongly right-skewed distributions (skewness = 2.29 and 2.83). A small number of wells in 2023 (*n* = 5) with very high As concentrations (>350 μg/L) disproportionately influence the mean, explaining the similarity between years and making the median a more representative indicator of typical groundwater conditions. A substantially higher fraction of wells with As < 5 μg/L was observed in 2023 (45%) compared to 2000 (29%), while the proportion of wells with As concentrations between 5−10, 10−50, and 50−100 μg/L was lower in 2023 (Figure 2d). The fraction of wells with very high As (>100 μg/L) remained similar between both time points (∼12%; Figure 2d). This shows, despite a reduction in median concentrations, the most hazardous wells remain prevalent.

Lower As was prevalent southwest of Hanoi (i.e., 21.0 °N, 105.8 °E), with fewer wells in 2023 containing As exceeding 50 μg/L (Figure 2b). Although some areas, particularly the southern part of the study area, show similar or even higher As levels in 2023 compared to 2000, the overall pattern indicates lower As in 2023. This suggests an overall decline in As across most of the study area over the past two decades. For aquifer-specific As patterns, see Figure SI8, Supporting Information.

### 3.2 Subregional As Contamination Patterns South of Hanoi (2000, 2013/14, 2023)

The southern and southwestern study area contained many of the highest As concentrations in 2000 (Figure 2a) and showed spatially variable concentrations by 2023 (Figure 2b). To examine temporal patterns within this high-As region, we used an intermediate 2013/14 data set covering part of the area. This data set covers a smaller subset of southern Greater Hanoi (20.90°N−21.02°N; 105.79°E−105.96°E) and was obtained from Nghiem et al.^46^ The 2013/14 survey area is relevant because previous studies documented substantial spatial variability in groundwater As across this region.^23,24^ The area is adjacent to the Red River, where sediment deposition and river-derived recharge can modify groundwater flow and redox conditions, promoting mobilization of As from recently deposited sediments.^23,24^ The analysis included all wells from the 2013/14 survey area (n = 137) and wells from the 2000 (*n* = 146) and 2023 (*n* = 141) data sets within this subset region (Figure 3a). This analysis compares broad As trends in this subregion rather than paired or well-specific changes, and does not represent the larger-scale 2000−2023 comparison.

**Figure 3:**
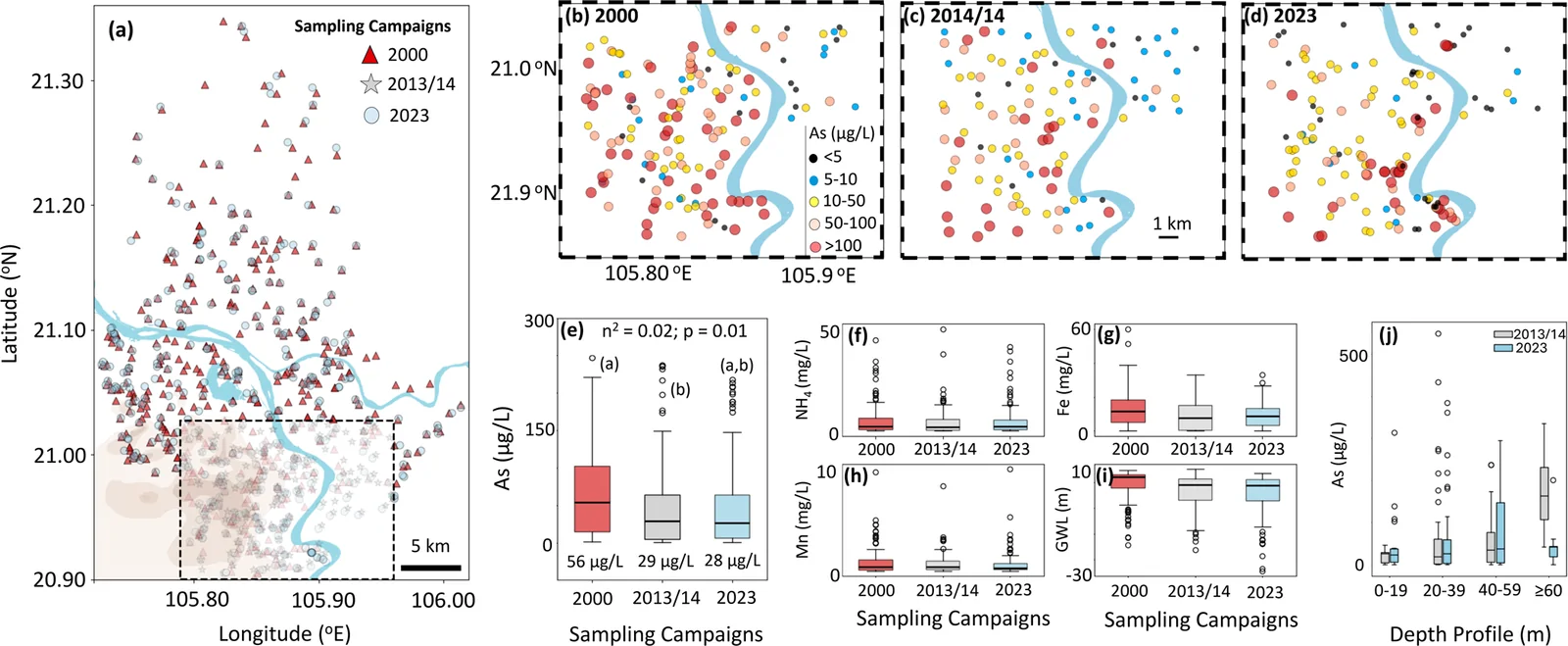
(a) Subregion of Hanoi comparing As concentrations between (b) 2000 (c) 2013/14, and (d) 2023. (e) Median As concentrations across the three campaigns; letters above each box represent statistical groupings based on the Conover-Iman test. (f–i) Median concentration of NH_4_^+^ (f), Fe (g), Mn (h), and GWL (i), respectively, across the three campaigns. (j) Median As levels stratified by well depth.

In 2000, many wells in this subregion exhibited high As concentrations exceeding 50 μg/L (Figure 3b). By 2013/14, As levels had declined, frequently falling below 10 μg/L, and remained similarly low in 2023 (Figure 3c,d). Median As levels declined significantly from 56 to 29.2 μg/L between 2000 and 2013/14 (*p* = 0.03; Figure 3e). However, As did not decline much further by 2023 (28.7 μg/L; *p* = 0.15; Figure 3e), suggesting that the main decline likely occurred before 2013/14. This pattern remained after accounting for spatial clustering with a 3 km spatial-block bootstrap: the 2000−2013/14 median difference was supported (−27.1 μg/L; 95% CI: −45.1 to −14.0 μg/L), whereas the 2013/14−2023 difference was not (−0.5 μg/L; 95% CI: −15.7 to 14.9 μg/L). Our comparison may thus indicate that a decline in As concentrations occurred before 2013/14; however, the precise magnitude and timing of the decline cannot be determined because the campaigns sampled largely different wells and may have differed in spatial coverage. We further assessed whether well type (private vs monitoring) systematically influenced temporal As patterns within the subregion. Groundwater As did not differ significantly between well types in either 2000 (Mann–Whitney *p* = 0.263) or 2023 (*p* = 0.270). A comparison was not possible for 2013/14 because all sampled wells were private domestic wells. Nevertheless, differences in well characteristics (e.g., well screen implementation) between private and monitoring wells remain a potential source of As concentration variability.

Moreover, NH_4_^+^ and Mn levels were stable across all three campaigns (5−6 mg/L; *p* = 0.48; Figure 3f; 0.30–0.39 mg/L; *p* = 0.50; Figure 3h). In contrast, Fe followed a similar trend to As, declining substantially from 12 mg/L in 2000 to 7 mg/L in 2013/14 (*p* < 0.01), with no significant change by 2023 (9 mg/L; *p* = 0.815; Figure 3g). An exploratory comparison of As concentrations by well depth between 2013/14 and 2023 suggests a decline in deeper wells (>60 m), with median As concentrations declining from 176 to 43 μg/L (Figure 3j), although this comparison is based on nine wells.

Groundwater levels declined between 2000 and 2023, with the steepest decline occurring between 2000 and 2013/14 (∼0.26 m/yr; Figure 3i). This decline, which may partially reflect groundwater pumping, coincided with lower As concentrations in 2023 relative to 2000 and is consistent with hydrological conditions that may promote groundwater replacement or flushing in some settings. Prolonged pumping and continued GWL decline can contribute to the formation of cones of depression, previously observed in the study area.^14,15,20^ These cones may modify hydraulic gradients and groundwater flow paths in ways that could enhance groundwater replacement and flushing processes in some hydrogeological settings, potentially contributing to lower concentrations observed in parts of the study area by 2023.

### 3.3 Groundwater Cones of Depression Evolution and Arsenic Patterns (2000−2023)

Given the observed changes in As concentrations, we next examine how the decadal evolution of groundwater cones of depression relates to groundwater As patterns. Groundwater cones of depression may influence recharge pathways and hydraulic gradients,^19^ potentially enhancing recharge infiltration and flushing processes.

In 2000, the Holocene cone of depression was shallow (∼−2 m a.s.l.), spatially limited, and exhibited a broad, low-gradient base (Figure 4a). Wells located within this cone had generally high As concentrations (>50 μg/L). By 2023, the cone had deepened (∼−7 m) and widened, with steeper slopes, resulting in a more pronounced cone of depression (Figure 4b). Holocene wells sampled in 2023 generally exhibited lower As concentrations (∼10−50 μg/L) than in 2000, although this pattern is based on a limited number of wells (*n* = 26). In 2000, the Pleistocene cone of depression was ∼−7 m deep and exhibited a broad, low gradient base (Figure 4c). Wells located within this cone had generally high As concentrations (>50 μg/L). By 2023, the cone had significantly deepened (∼−22 m) and widened, presenting a narrower cone base (Figure 4d). Pleistocene wells sampled in 2023 exhibited lower As concentrations (<5 μg/L), except along shallow marginal cone edges where As concentrations can remain high (>50 μg/L). The study by Winkel et al.^20^ hypothesized that in the Greater Hanoi region, groundwater pumping had exacerbated As contamination by drawing down As and/or As-mobilizing compounds from contaminated Holocene to deeper Pleistocene aquifers. The current study indicates that the existing cones deepened over time, developing steeper vertical and lateral hydraulic gradients. These conditions may be consistent with enhanced throughflow and recharge infiltration, which could promote groundwater replacement, dilution, and flushing of dissolved As in some hydrogeological settings. One hypothesis is that high As concentrations along cone margins may be explained by weaker hydraulic gradients and longer residence times relative to the cone interior, facilitating the persistence of biogeochemically mobilized As. This is consistent with studies showing groundwater age and flushing history can influence As distributions.^30,32^ Groundwater age and residence time data were unavailable, so this hypothesis could not be tested directly; however, our findings motivate assessing whether the prominence of redox-related and hydrological predictors differed between the 2000 and 2023 data sets.

**Figure 4:**
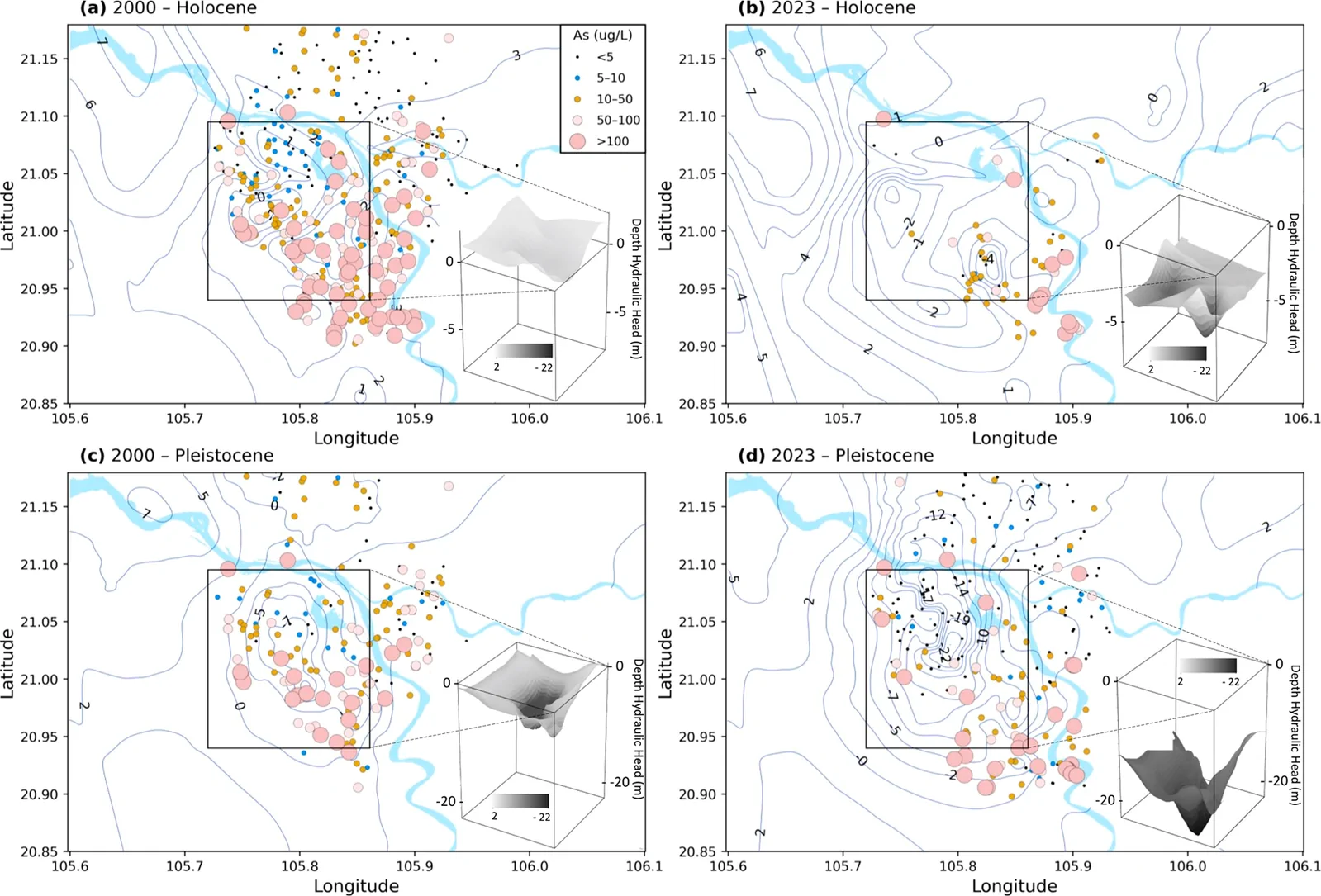
Evolution of groundwater cones of depression for (a) Holocene aquifer (2000), (b) Holocene aquifer (2020), (c) Pleistocene aquifer (2000), and (d) Pleistocene aquifer (2020). Black boxes delineate cone boundaries; corresponding 3D zoomed views are shown in the lower right of each panel. The gray scale bar indicates the depth below sea level.

### 3.4 Identifying the Key Predictors of As Contamination (2000−2023)

For the full study area, we examined whether the relative importance of redox-related and hydrological predictors differed between 2000 and 2023 using wells with available depth information (*n* = 461 in 2000; *n* = 344 in 2023). We selected concentrations of NH_4_^+^, Mn, and Fe as representative redox proxies, and seasonal mean GWL as a hydrological indicator (Section 2.5.2 for rationale). SHAP assessed predictor importance within separately trained models; data set comparisons were limited to within-model predictor rankings. DML separately evaluated associations between individual predictors and As concentrations.

For 2000, the CatBoost model achieved a strong predictive performance (train *R* ^2^ = 0.75; test *R* ^2^ = 0.70; test RMSE = 62.9, test bias = 8.35%). For 2023, performance was slightly stronger (train *R* ^2^ = 0.84; test *R* ^2^ = 0.79; test RMSE = 60.4; test bias = 7.30%). Spatial autocorrelation was assessed using 5-fold spatial-block cross-validation, with wells from the same 3 km block retained in the same fold. For 2000, mean cross-validation *R* ^2^ decreased from 0.604 under random cross-validation to 0.601 under spatial-block cross-validation, while RMSE increased from 45.00 to 46.37 μg L^−1^. For 2023, mean *R* ^2^ decreased from 0.75 to 0.72, while RMSE increased from 53.17 to 54.60 μg L^−1^. Overall, spatial autocorrelation caused limited inflation of model performance at the 3 km scale and did not alter feature rankings, although the modest *R* ^2^ reduction in 2023 indicates some spatial influence on predictive performance. Additional model robustness and validation analyses supported the reliability of both CatBoost models (Supporting Information 3, Sections 3.4; Figures SI1−SI3; Tables SI1−SI6).

In 2000, NH_4_^+^ and Fe were the most important model variables influencing predicted As concentrations, with mean SHAP scores of 35.1 and 27.4 (Figure 5a). Double/debiased machine learning indicated a statistically significant positive association between Fe and As concentrations (*p* = 0.012; θ = 0.48; 95% CI [0.11, 0.87]), showing that higher Fe concentrations were associated with higher As concentrations. Groundwater level (absolute value) ranked lower in the SHAP analysis, with a mean absolute SHAP contribution of 12.8. Although lower GWL was associated with lower As concentrations, this relationship was statistically insignificant (*p* = 0.544; θ̂ = −1.37; 95% CI [−5.83, 3.07]). In 2000, predictors related to redox conditions were strongly associated with As concentrations, whereas GWL contributed comparatively less within the model framework.

**Figure 5:**
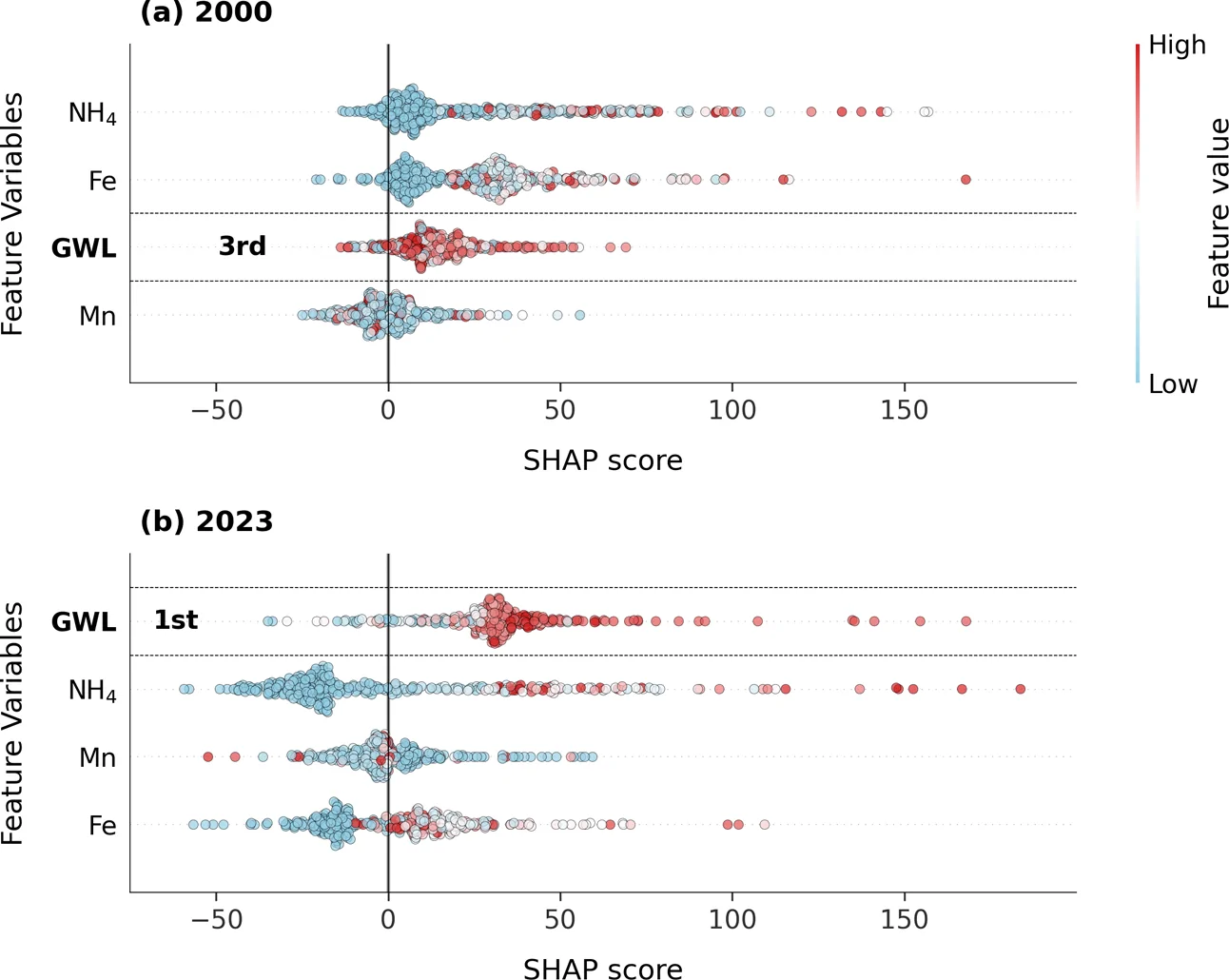
SHAP distributions and within-model rankings for GWL, NH_4_^+^, Fe, and Mn in independently trained 2000 (a) and 2023 (b) models. Blue and red dots indicate low and high feature values of As, respectively. Positive SHAP scores indicate increased model-predicted As concentrations; negative scores indicate decreases. SHAP values are model-specific; absolute magnitudes should not be compared quantitatively between panels.

In the 2023 model, GWL ranked highest of four predictors by mean absolute SHAP importance; lower GWL was associated with lower predicted As (Figure 5b). It should be noted that SHAP comparisons between data sets (i) are limited to within-model predictor rankings and ranking differences may reflect data set-specific predictor covariance rather than changes in processes controlling As and (ii) should be interpreted as model-inferred predictor importance, not evidence of causal mechanisms. Still, it is interesting that GWL had a greater model-inferred relative importance in the 2023 model than in the 2000 model. The association between lower As and lower GWL in the 2023 data set was statistically significant (*p* < 0.001; θ̂ = −12.48; 95% CI [−18.52, −6.44]).

Stratifying by aquifer, the association between lower GWL and lower As was stronger in Holocene (mean SHAP = 13.9 [2000; *n* = 257] and 31.4 [2023; *n* = 70]) than in Pleistocene aquifers (mean SHAP = 12.8 [2000; *n* = 204] and 28.3 [2023; *n* = 274]; Figure SI10, Supporting Information). Holocene aquifers may be more susceptible to groundwater replacement and flushing processes associated with pumping-induced hydraulic gradients, potentially due to their greater hydraulic connectivity to surface recharge zones such as rivers and lakes. The SHAP scores for the two aquifers were not significantly different within either campaign. This is unsurprising, as both the Holocene and Pleistocene aquifers experienced a comparable average GWL decline between 2000 and 2023 (3.5 m, 2.9 m, respectively; Figure SI11, Supporting Information). Because aquifer classification may influence hydrogeological conditions and GWL−As relationships, sensitivity analyses were conducted separately for Holocene and Pleistocene aquifers. Full results are provided in Supporting Information (Methods: Section 3.1).

### 3.5 Influence of Groundwater Level Changes on As Concentrations

To further examine the relationship between GWL change and As concentrations, we compared As levels in wells sampled during the dry season in 2023 with and without significant GWL declines (*n* = 251; Figure 6a,b). Almost all wells sampled during the wet season exhibited significant GWL declines, meaning such comparisons could only be made for wells sampled during the dry season. Specifically, the wet-season data set contained too few wells without significant GWL declines to provide a meaningful comparison group.

**Figure 6:**
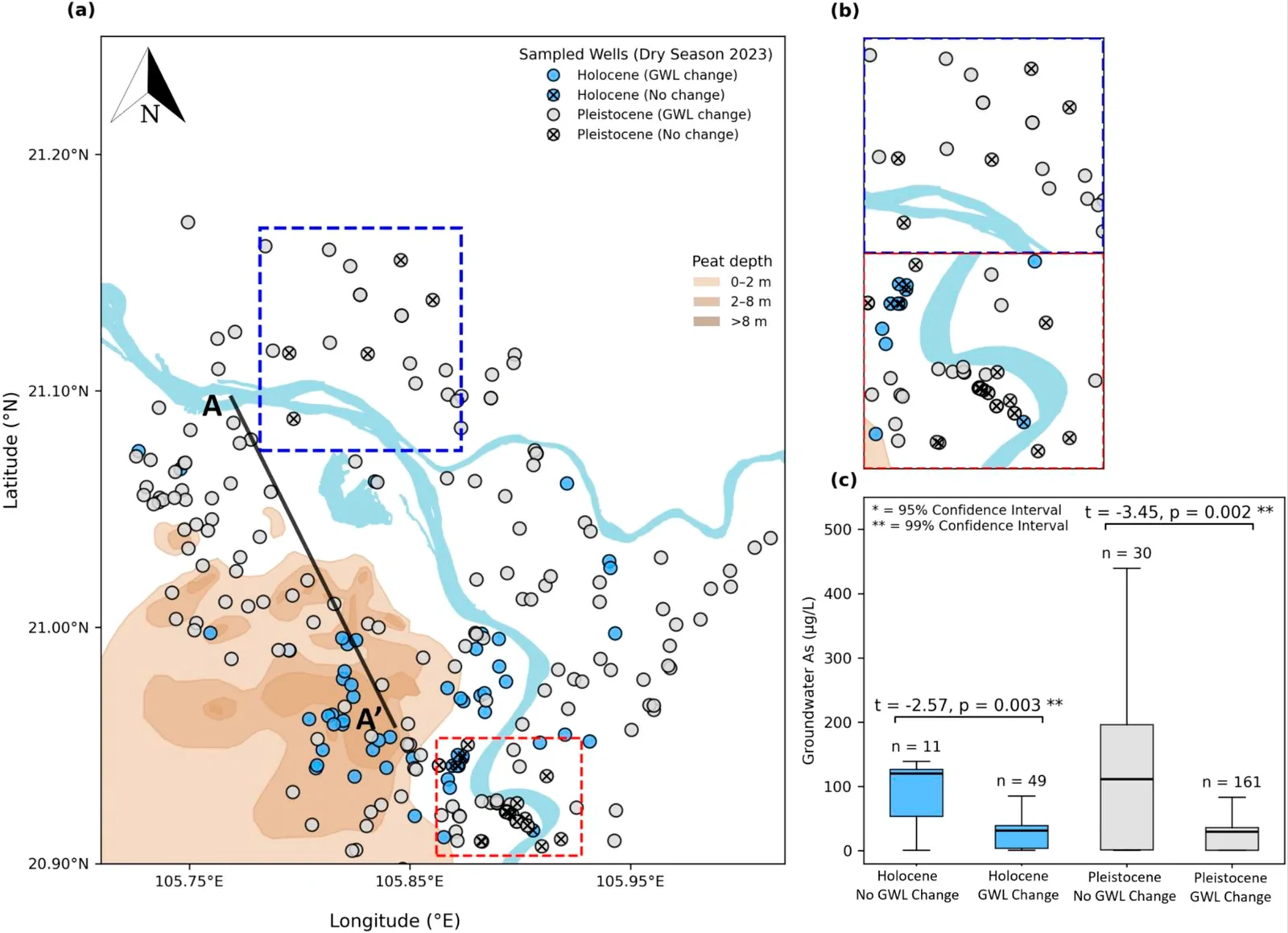
(a) Wells showing significant GWL changes (open circles) and no change (*x*-marked circles) in Holocene (blue) and Pleistocene (gray) aquifers. The black line (A−A′) indicates the location of the schematic cross-section show in Figure 7. (b) Zoomed-in view of wells without significant GWL change. (c) Median As concentrations in Holocene and Pleistocene wells with and without significant GWL change. Reported *z* and *p* values are from a Wilcoxon test.

Among wells tapping into the Holocene aquifer, median As concentrations were higher in wells without GWL declines (*n* = 11; As = 120 μg/L) than in those with declines (*n* = 49, As = 31.4 μg/L; Figure 6c), with the difference being significant (*z* = −2.57, *p* = 0.003). For wells tapping the Pleistocene aquifer, median As concentrations were higher in wells without GWL declines (*n* = 30; As = 111 μg/L) than in those with declines (*n* = 161; As = 29.2 μg/L), also a significant difference (*z* = −3.1, *p* = 0.004). After accounting for spatial clustering using cluster-robust regression with 3 km blocks (Section 2.6), these differences remained significant. Specifically, Holocene wells that experienced GWL declines had, on average, 88.6 μg/L lower As concentration than those without GWL declines (*p* = 0.022; 95% CI: −163.3 to −14.0 μg/L). Similarly, for Pleistocene wells, those that experienced GWL declines had, on average, 82.0 μg/L lower As concentration than wells without GWL declines (*p* < 0.001; 95% CI: −118.0 to −46.1 μg/L).

We examined whether the association between GWL decline and lower As concentrations in these wells varied across hydrochemical settings using linear regression (Table 2). The GWL−As association was negative and significant (β = −6.20, *p* = 0.0007; Table 2), indicating lower As in wells experiencing greater GWL declines. We tested whether the GWL−As association was modified by hydrochemistry. Only CH_4_ showed a significant interaction: wells with higher CH_4_ levels exhibited a weaker negative association between GWL decline and As levels (β = 9.74, *p* = 0.0005; Table 2). High-CH_4_ wells generally experienced smaller GWL declines; wells above the median CH_4_ concentration showed lower GWL declines than those below it (Figure SI12, Supporting Information). CH_4_ itself may not directly weaken the GWL−As association; instead, elevated CH_4_ likely reflects wells that experienced smaller GWL declines and reduced flushing. High CH_4_ levels may also be associated with methanogenic conditions under which Fe­(III) oxides undergo dissolution, releasing As into groundwater.^24,47^ In such environments, ongoing As release may counteract the removal of dissolved As through hydrological flushing, further weakening the association between GWL decline and lower As concentrations. Elevated CH_4_ has been shown in Van Phuc region of the study area to promote free gas formation within the aquifer, reducing hydraulic conductivity and groundwater flow, which may limit As flushing.^62^

**Table 2:** (a) Main Relationship between GWL Change and As Levels; (b) Interaction Effects of Hydrochemical Covariates (CH_4_, Fe, Mn, NH_4_^+^, Na, DOC, SO_4_) on the GWL−As Slope^a^

model component	variable	estimate	95% CI	*P*-value	interpretation
(a) main relationship As ∼ΔGWL	ΔGWL	−6.20	−9.77 to −2.65	0.0007*	significant: ↓ GWL = ↓ As
(b) covariate modifiers of the As ∼ΔGWL slope	CH_4_	9.74	4.27–15.2	0.0005*	significant
	Fe	2.29	−0.57–5.14	0.116	not significant
	Mn	−1.40	−6.32–3.52	0.576	not significant
	NH_4_^+^	4.93	−3.81–13.7	0.267	not significant
	Na	−2.37	−5.53–0.79	0.141	not significant
	DOC	−7.83	−16.5–0.78	0.074	not significant
	SO_4_	0.67	−2.64–3.98	0.689	not significant

a Covariates are listed in the order they were entered into the regression model. Positive estimates indicate a weakening of the negative GWL−As association; negative estimates show the opposite. Asterisks show statistical significance.

### 3.6 Implications of Research Findings

While using two independent, unpaired data sets (2000 and 2023) precludes tracking well-specific trends or annual dynamics, we demonstrate an overall decrease in median arsenic concentrations in 2023, particularly in the cones of depression. We therefore hypothesize that the continued evolution of groundwater depression cones under sustained pumping may have promoted groundwater replacement, flushing, or dilution, contributing to lower As concentrations in parts of the study area. CatBoost and SHAP results suggest that redox-related predictors were more influential in 2000, whereas GWL had greater relative importance in 2023, consistent with a possible shift from stronger redox influence toward greater hydrological control. To further verify this hypothesis, groundwater age tracers, solute mass balance and direct pumping data would be valuable.

We suggest an evolution of As contamination patterns over time in our study area. Although As contamination of the Hanoi aquifers was only discovered shortly before 2000 ^44^ elevated NH_4_^+^ concentrations in the 1990s indicate weaker groundwater flow and strongly reducing groundwater conditions in this period.^45^ Likely, weaker groundwater flow during this period favored redox-driven As mobilization^45^ (Figure 7, first panel). The contamination pattern observed in 2000 reflects an early stage in the evolution of the cone of depression (Figure 7, second panel). At this phase, sustained pumping likely caused the downward migration of As or As-mobilizing compounds from Holocene into Pleistocene aquifers, as discussed in Winkel et al.^20^ The present study shows that continued pumping and deepening depression cones coincided with lower As concentrations, which can potentially be explained by dilution and flushing as possible contributing processes (Figure 7, third panel). This scenario may indicate that in these areas As supply, e.g., via (re)­mobilization does not keep pace with flushing; however, this hypothesis requires further investigation.

**Figure 7:**
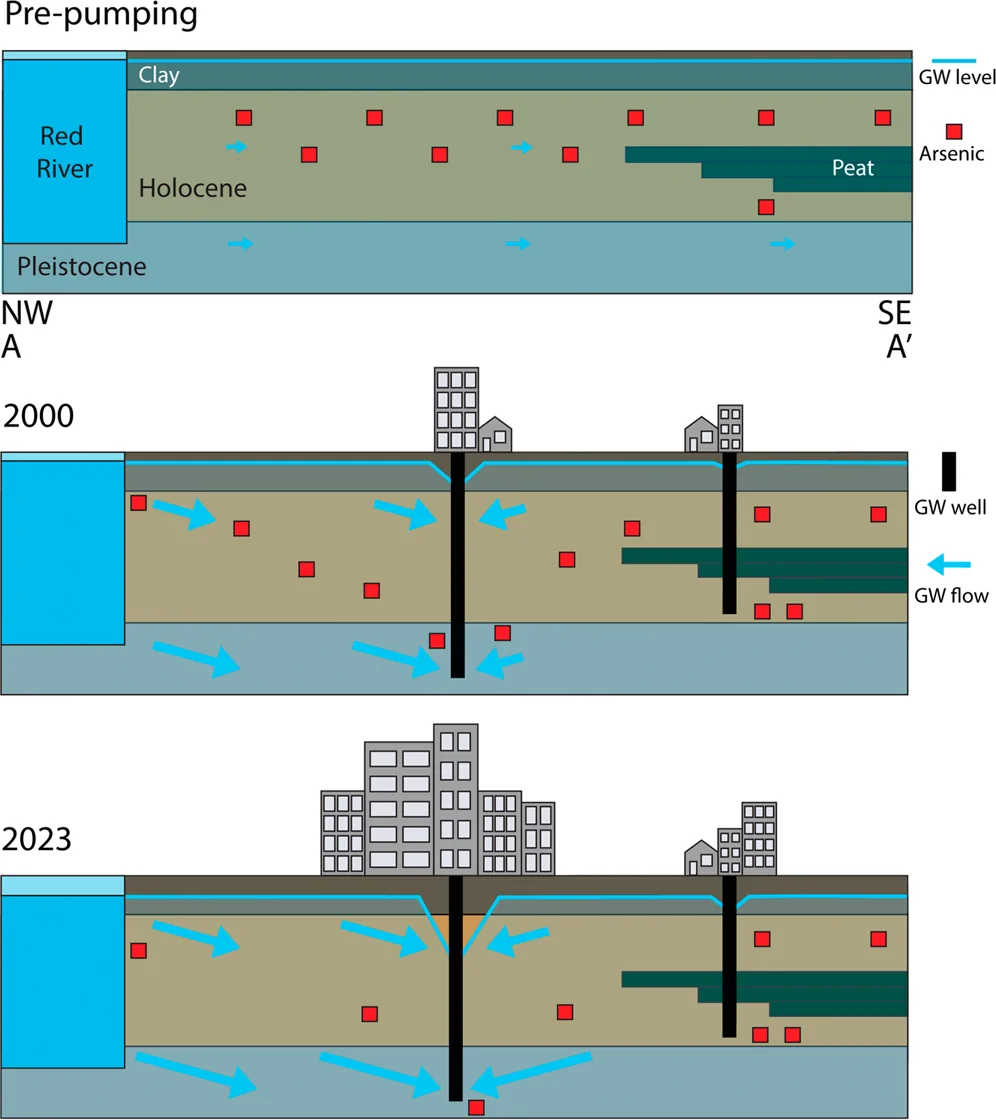
Schematic cross-section illustrating the gradual flushing and decline of As in groundwater under sustained pumping in the Red River Delta (vertically and horizontally not to scale; location shown in Figure 6a, transect A−A′). Red squares indicate elevated groundwater As concentrations (>10 μg/L). The top panel represents prepumping conditions, where groundwater As concentrations were high and flow was weak, largely undisturbed, and directed from northwest to southeast. The middle panel represents early stage pumping (ca. 2000), with the formation of shallow cones of depression. Early stage pumping and the initial development of these cones of depression drive the gradual drawdown of As from shallow Holocene sediments into the deeper, previously uncontaminated Pleistocene aquifer (Winkel et al., 2011). The bottom panel illustrates how intensified and sustained pumping (ca. 2023) deepens the cones of depression, enhances hydraulic gradients, and increases flushing, which may contribute to lower As concentrations.

Our interpretation is not only consistent with previous studies showing that groundwater abstraction can initially advect As from contaminated aquifers to previously uncontaminated aquifers, but also with studies showing that pumping can lead to As flushing. Hoang et al.^26^ showed that sustained pumping and the formation of large depression cones in parts of the Red River Delta can alter hydraulic gradients. Their study further emphasized that these processes are strongly controlled by local hydrogeological conditions. However, as is also pointed out by Hoang et al.^26^ and other studies (e.g., Van Geen et al.^19^ and Nghiem et al.^46^), sustained pumping promotes riverbank infiltration, which can mobilize As concentrations close to the river. This agrees with our study, indicating that As concentrations close to the river have generally not declined, although our data set only contains a limited number of wells near rivers. Furthermore, As concentrations in the southern part of the study area remain high, potentially indicating ongoing As mobilization from organic-rich sediments at depth, such as peat layers, needing further research.

Potentially, our findings have implications for groundwater management in other regions with As contaminated aquifers as they may generalize to permeable, hydraulically connected aquifers receiving low-As recharge with limited As replenishment from sediments. However, these effects are strongly context dependent. Patterns in Hanoi should thus generally not be considered representative of other As-affected aquifers. Conversely, these findings may not generalize to settings where pumping redistributes As or As-mobilizing compounds into previously less contaminated aquifers, as observed in Winkel et al.^20^ and van Geen et al.,^19^ and in Bangladesh.^17,28,29^ Furthermore, pumping can reduce groundwater availability^65^ and contribute to land subsidence.^63^ The association between groundwater level decline and lower As concentrations should thus not be interpreted as evidence that pumping is a viable As-mitigation strategy.^64^

Finally, long-term changes in As patterns are governed by additional complex factors, including local well lithology and the replenishment of As from riverine, aquifer, and aquitard sediments. While evaluating these components was outside the scope of this study, resolving the main mechanistic uncertainty requires quantifying the mass balance between As inputs and its removal via flushing and dilution. Incorporating information on particulate phase As alongside hydrochemical data and groundwater age or residence time indicators will be critical to compare sediment-driven mobilization with these removal mechanisms.

## Supplementary Material







## Notes

Additional methods, machine-learning model evaluation, supplementary hydrochemical and hydrological analyses (XLSX)
Figures, and tables provided in the supplementary data set (PDF)
Field and laboratory blanks showed no anomalies (PDF)
