# Supplementary material for: Modeled Groundwater Decline is Associated with Lower Arsenic Concentrations in Hanoi
Source: Environ Sci Technol. 2026 Oct 18;60(43):30887–900. doi: 10.1021/acs.est.6c07700 (PMC13738782; doi:10.1021/acs.est.6c07700)
Supplement: Supplementary file 1 [file es6c07700_si_002.pdf]

Supplementary Information for

Modeled Groundwater Decline is Associated with Lower Arsenic Concentrations in Hanoi

David Haaf[1,2], Michael McGlade*[1,2], Athena A. Nghiem[1,2,3], Duyen Thi Vu[4], Tobias Steinert[1,2], Viet Hung Pham[4], Linh Khanh Bui[5], Thanh Ngoc Tong[6], Duong Du Bui[5], Andrea Stenke[1,2], Michael Berg[1], Lenny H. E. Winkel*[1,2]

[1] Eawag: Swiss Federal Institute of Aquatic Science and Technology, Department of Water Resources and Drinking Water, 8600 Dubendorf, Switzerland.

[2] Institute of Biogeochemistry and Pollutant Dynamics, Department of Environmental Systems Science (D-USYS), ETH Zurich, 8092 Zurich, Switzerland.

[3] Department of Geoscience, University of Wisconsin-Madison, Madison, WI 53706, USA.

[4] Key Laboratory of Analytical Technology for Environmental Quality and Food Safety Control (KLATEFOS), VNU University of Science, Hanoi 11417, Vietnam.

[5] Water Research Institute, National Center for Water Resources Planning and Investigation (NAWAPI), Hanoi 10000, Vietnam.

[6] Vietnam Water and Environment Association, Hanoi 11307, Vietnam.

* Corresponding authors: mmcglade@ethz.ch, Lenny.Winkel@eawag.ch

Supplementary Information 1: Data Quality Check

pH Measurements

Issues arose with some pH recordings in the 2000 dataset. Since As is commonly released under reducing conditions by Fe(III) reduction, which consumes H^+ ions and tends to raise pH (Borch et al., 2010), elevated As is often, but not exclusively, associated with higher pH (Postma & Jakobsen, 1996). The 2000 dataset does not follow this general pattern, showing high As concentrations for wide pH ranges between 4 and 7.5. Arsenic can be released under iron sulfide oxidation, especially at low pH; however, the high Fe (mean = 11.2 mg/L) and NH_4 (mean = 7.9 mg/L) concentrations observed in 2000 indicate reducing rather than oxidizing conditions. This disparity may reflect pH probe calibration issues.

EC Measurements

We identified issues with electrical conductivity (EC) measurements in the 2000 campaign. We estimated the minimum expected conductivity (EC_min) from alkalinity and Ca-Mg concentrations using Equation 1 (Marion & Babcock, 1976), with μ as the ionic strength calculated in PHREEQC.

$$EC\_min = 6.67 \times 10^4 \times \mu^{0.991} \qquad \text{(Eq.1)}$$

Alkalinity was selected because it was measured by automated laboratory acid titration, providing a robust benchmark for evaluating EC measurements. Calcium and Mg were not measured in 2000, so their contribution was approximated using the mean Ca:Mg ratio derived from the 2023 dataset. The Ca:Mg ratio showed limited dispersion around its mean (2.47), supporting the use of an average Ca:Mg ratio in EC_min calculations. Many wells sampled in 2000 had EC values below EC_min, which is geochemically impossible because conductivity cannot be lower than the charge needed to balance alkalinity (Marion & Babcock, 1976). In contrast, the other campaigns aligned well with EC_min, indicating that the 2000 EC probe was likely also mis-calibrated.

Eh Measurements

Given the anomalous pH and EC measurements, we assessed the reliability of Eh. In both the 2000 and 2023 datasets, elevated aqueous Fe (presumably Fe^{2+}) occurs at Eh > 50 mV. This behaviour is consistent with established evidence that redox zonation in groundwater is not strictly sequential and that Fe(III) reduction can occur under positive Eh conditions (Postma & Jakobsen, 1996). However, Eh measurements were excluded from further analysis because, like pH and EC, they were obtained using field probes.

Supplementary Information 2: Hydrological MODFLOW Modelling

The hydrological dataset used in this study encompasses monthly groundwater level, velocity and flow direction information between 1995 and 2020 provided by NAWAPI. The groundwater level, velocity and direction were modeled by NAWAPI with Visual MODFLOW and MODFLOW based on 57 Holocene observation wells and 62 Pleistocene observation wells all over the Red River delta with a spatial resolution of 250 x 250 m for Greater Hanoi and 1 x 1 km for the delta based on a model grid of 108 rows and 142 columns over an area of 12,290 km² from 20.08°N 151 - 21.35°N and 105.27°E - 107.41°E. The surface terrain for the MODLFOW model was built based on elevation points of the 1:50,000 topographic map of Vietnam and the measured elevation at the monitoring wells. The vertical model domain includes 5 depth layers with the (i) weakly permeable surface layer, (ii) the unconfined Holocene layer, (iii) the Holocene to Pleistocene water insulation layer (aquitard), (iv) the confined Pleistocene layer, and the (v) fractured Neogene layer. The top, depth and thickness, as well as the hydraulic conductivity parameters of the 5 layers are based on hydrogeological strata from boreholes combined with circa 1000 drills for pump tests throughout the delta.

The pump tests were conducted with a constant and stable flow rate including single and multiple wells including pumping durations at least 5 times longer than the calculated value for the water movement to reach near-steady state. The groundwater recharge was set to 5–161, 8% of the total monthly average precipitation measured at 11 weather stations throughout the delta and further subdivided by Thiessen polygons. The quantified evapotranspiration information was also calculated from data obtained at the same weather stations. Furthermore, groundwater pumping data from production wells as well as household wells, which were grouped on commune scales, were collected and integrated as averaged quarterly pumping rates in the model. The main rivers (the Red River, Day River and Ca Lo River), large lakes as the West Lake in Hanoi and the sea were used as general head boundaries for Holocene unconfined aquifer (HUA) and Pleistocene confined aquifers (PCA), while the bedrock was defined as a depth boundary with a groundwater flow of 0. The initial conditions are based on the conditions in January 1995 of overall monitoring wells. The model was validated on the steady flow conditions in January 1995 and unsteady flow conditions between January 1995 and December 2020. The steady and unsteady flow conditions showed good validation results for the groundwater level (Steady Flow: ME = 173 0.46 m, MAE = 1.07 m, RMSE = 1.5 m, rRMSE = 5.7 %; Unsteady Flow: MEHUA = 0.197 m, MAEHUA = 0.861 m, rRMSEHUA = 5.9 %; MEPCA = 0.067 m, MAEPCA = 0.906 m, rRMSEPCA = 175 2.621 %).

Based on the modeled groundwater levels, flow direction and flow velocity in m/day were calculated inside Visual MODFLOW. These parameters were provided as raster data by NAWAPI. The provided hydrological raster's were used to estimate the flow from the wells over time, starting at the time point for hydrochemical sampling. First, the flow distance and direction was calculated for the sampling month and year. The flow distance was limited to the distance the water would flow in 30 days in the estimated direction. The endpoint of this flow path was used as the starting point for the next section of the flow path, which used the flow direction and flow velocity of the previous month. If the monthly

flow distance intersected with multiple MODFLOW raster cells, the distance and the time (in days) of the flow inside the first MODFLOW cell was estimated. The distance of the flow path in the next adjacent MODFLOW cells was estimated for the remaining days (30 days - days in previous cells) of the flow path. If needed, this was repeated for the next adjacent MODFLOW cells until the 30-day mark was reached. These steps were repeated for each available month and year with hydrological information from the provided MODFLOW results.

However, because the wells were sampled in different years, the estimation was limited for each flow path to a maximum of 8 years, which is the time period between the earliest sampled wells in 2002 and the start of the MODFLOW model in 1995, in order to get more comparable estimates between the wells (i.e., for wells sampled at different time points). This means that if e.g., a well was sampled for hydrochemical analysis in January 2008; the flow path was estimated from this month and year until January 2000. This method of estimation of the horizontal flow path was chosen over MODPATH or other particle tracking models due to limited access to the specific MODFLOW files from the original model to perform such particle-tracking analyses. Overall, the complete horizontal flow paths over a maximum of 8 years ranged between 0.12 m to 5794 m with an average of 666 m ± 798 m (mean ± standard deviation).

Next, the flow paths were intersected with the raster cells of the geology model. Since the flow paths mainly represents flow in horizontal directions and the investigated hypothesis refer mainly to sediment at the wells and directly above the wells, the geology over the flow path was constrained in vertical directions. These vertical restrictions of the geology of the flow path follow the definitions of the screen and above screen layers at the well, including for the geology layers of the flow distance a "layer of defined well screen" and a "layer above". This means that for each raster cell stack intersecting with the flow path, the layer of defined well screen is defined vertically from the well depth until the next change in sediment composition in vertical direction and the layer above from the point in sediment composition change until the next sediment change. As for the screen at the well, the layer of defined screen was also limited to a maximum of 10 m in thickness. Since the flow path includes temporal information, for each of these stack of raster cells, the month and year of the respective part of the flow path was allocated. Based on this temporal information, the geological raster stacks were horizontally combined to yearly time steps, starting from the month of sampling to the end of the same year, and each subsequent step backwards contains a full year. In this step, the sediment compositions of each vertical layer were combined and the thickness of the vertical layers was averaged.

Based on the lateral and vertical constraining of the flow path geology, 32 geological predictors, representing the hydrogeological conditions of the flow path, were obtained for every well, i.e., 8 sediment composition descriptions and 8 thickness values (in m) of the "layer above" along the flow path, 8 sediment composition descriptions and 8 thickness values (in m) of the "layer of defined well screen" along the flow path.

Supplementary Information 3: CatBoost Audit Evaluation

1. Introduction

Ensuring the reliability of machine learning models to produce accurate results is important given their "black-box" nature. Unlike "white-box" models such as linear regression, where the underlying analytical mechanisms are easily understood, the internal workings of "black-box" models around how they analyse and learn data must be appreciated when training and developing such models. This mini-paper rigorously examines the (i) generalisation, (ii) overfitting, (iii) robustness, and (iii) stability of our two CatBoost machine learning models used to rank variable importance using SHAP for the 2000 and 2023 datasets. We provide a rigorous comparison of the performance metrics, evaluating their suitability for our variable importance analysis.

2. Methods

2.1 Purpose and Overview

To reiterate, our purpose was to rigorously assess whether the machine learning model can accurately and reliably rank feature importance. A step-by-step outline of this evaluation workflow is presented in Figure SI1 below.

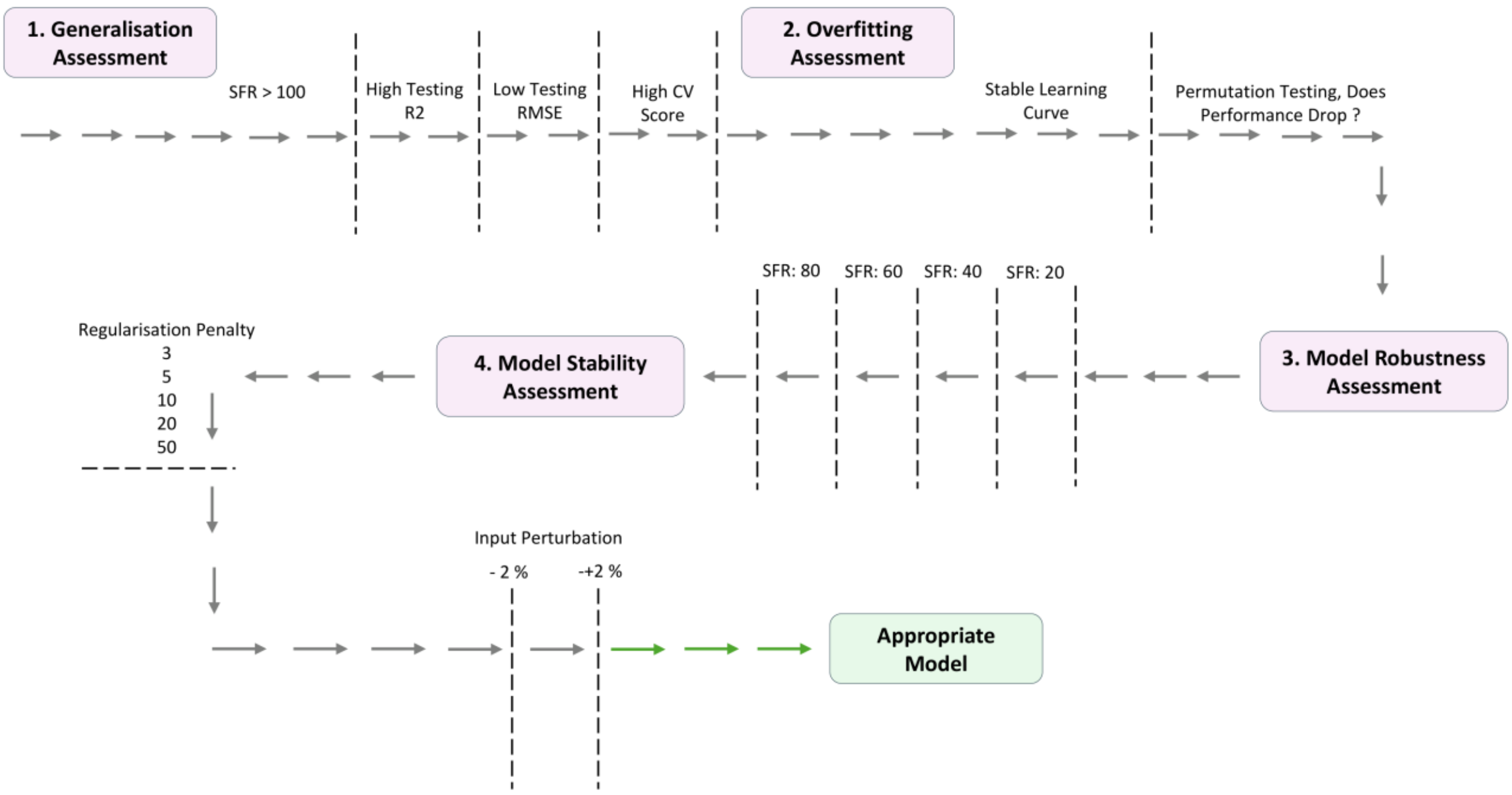

Figure SI1. Workflow for evaluating the (i) generalisation, (ii) overfitting, (iii) robustness, and (iv) stability of our CatBoost models. Throughout this mini-paper, our aim is to follow through this process, continuously evaluating and critiquing our model performance, assessing its overall suitability for estimating variable importance (main paper).

2.2 Machine Learning and Linear Regression Model Training/Generalisation Assessment

We trained a CatBoost, random forest, and linear regression model to evaluate which model most accurately predicts and ranks the hydrochemical variables associated with As groundwater contamination in 2000 and 2023 datasets. Our target variable was As with four hydrochemical features: NH_4, GWL, Fe, and Mn. The 2000 dataset contained 461 samples and 2023 dataset had 344 samples. The sample-to-feature ratio (SFR), which defines the number of observations divided by the number of

predictor features, for the 2000 and 2023 datasets, was 117 and 85, respectively. Zhu et al., (2023), who propose a best-practice workflow for machine learning in environmental science, suggests a SFR of at least 10 (i.e., 10 rows of data per feature variable) is considered the minimum for ensuring model stability, while values exceeding 100 are considered excellent. One our datasets exceed this excellent threshold, while the other dataset exceeded well above the minimum threshold.

CatBoost and random forest allow a comparison of “boosted” vs. “bagged” learning approaches. Boosting iteratively combines weak decision-tree learners, i.e., learners slightly better than random, into stronger learners (Bentejac et al., 2021). Bagging (bootstrap aggregation) trains multiple decision trees simultaneously on different subsets of the dataset, and then averages their predictions to reduce variance (Bentejac et al., 2021). CatBoost was selected over other boosting algorithms (e.g., XGBoost, LightGBM) because it handles variable interactions efficiently and is less sensitive to hyperparameter tuning with small datasets. Regression-based models perform well on small datasets, requiring few tuning parameters, and a simple linear decision boundary helps reduce variance and overfitting, meaning such models have been widely used for groundwater As estimations (Cao et al., 2018; Iqbal et al., 2023; Winkel et al., 2011). We chose linear regression because our target variable As was continuous.

Random forest and linear regression models were trained using the *scikit-learn* package, and CatBoost using the *catboost* package in Python (Version 3.13.5). We trained such models separately for the 2000 and 2023 datasets. Training and testing splits were established using 5-fold cross-validation. Overfitting was examined using learning curves, cross-validation, and permutation testing (i.e., shuffling the target feature; Table SI1). These models were hyperparameter tuned using systematic grid searches. Performance was evaluated by analysing the testing and training error (RMSE, MAE), and accuracy (R^2). For information around the specified tuning metrics, test-train splitting ratios, and model performances, see Tables SI2-4. Five-fold cross-validation evaluated model stability across different dataset folds, determining whether performance was consistent rather than driven by chance. We compared various performance metrics across different folds, including R^2, RMSE, and MAE.

2.3 Overfitting Assessment

We assessed whether the selected machine learning model minimises overfitting irrespective of the small training dataset size. We examined overfitting using two techniques: (ii) learning curves and (ii) permutation testing:

(i) Learning curves

Learning curves evaluated model performance under varying dataset sizes (e.g., 50, 100, 150, 200). For each increment of dataset size, we evaluated training and testing performances, aiming to diagnose which dataset size leads to overfitting. A large gap–where training performance remains high but testing performance decreases–indicates overfitting. Moreover, significant fluctuations in either training or testing performances with small increments in dataset changes is another indicator of overfitting.

(ii) Permutation Testing

Permutation testing, which randomly shuffles the target variable (As), while keeping the predictor features fixed (GWL, Mn, Fe, and NH_4), evaluated whether the machine learning model was learning meaningful relationships and not memorising noise. Essentially, after shuffling, if the model performance collapsed, we conclude the model was learning a real relationship. Alternatively, if the performance remained high this suggests it was “cheating” by picking up artefacts or noise (data leakages). This procedure was repeated 100 times (x 100 shuffles) and we computed the average testing and CV performance.

2.4 Model Robustness Assessment

We evaluated model robustness by examining performance across progressively reduced SFR thresholds (e.g., < 10, 10, 20, 30…). This analysis identified the minimum dataset size at which the model can still perform reliably without overfitting. The original SFR values for the 2000 and 2023 datasets was 117 and 85, respectively; however, progressively reducing this ratio enabled us to evaluate how small the dataset can become before the model can no longer discern meaningful patterns and overfits the data.

We assessed model performance across different SFR thresholds by comparing testing R^2, RMSE, and MAE. The dataset was iteratively subsampled (i.e., reducing the number of observations while retaining the number of predictor features). Uniform random sampling was used so that all observations had equal probability of being retained or removed, mitigating sampling bias. A fixed random seed was applied for reproducibility. A significant drop in predictive accuracy or a large increase in error metrics was used to identify the point at which the model became unstable. For each incremental reduction in SFR, learning curves were also used to evaluate overfitting risk. For example, if performance suddenly drops between SFR 60 and SFR 50, this indicates that a SFR of 50 is insufficient to support stable model performance.

2.5 Model Stability Assessment

We evaluated performance stability under varying stress scenarios using two forms of perturbation: (i) regularisation and (ii) input sensitivity testing. The purpose of these tests was to stress-test the model, evaluating its stability performance under controlled constraints.

(i) Regularisation Robustness

The first stress test examined regularisation sensitivity by applying different regularisation strengths to the model. If predictive performance declined significantly (e.g., a drop in testing R^2 or a rise in RMSE or MAE) under weak (L2 = 3) or sever (L2 = 50) regularisation, this indicated poor generalisation stability. For random forests, regularisation was applied by adjusting *max_depth* (very weak = none, weak = 20, moderate = 10, strong = 7, very strong = 5). For CatBoost, we adjusted *l2_leaf_reg* (very weak = 3, weak = 5, moderate = 10, strong = 20, very strong = 50).

(ii) Input Sensitivity Robustness

The final stress test assessed prediction stability under small perturbations to the input features. Hydrochemical variables (Fe, Mn, NH_4) and hydrological variables (GWL) inherently contain noise; a robust model should retain predictive accuracy despite this noise. Each input feature was perturbed simultaneously by -2% and +2%, and changes in performance (R^2, MAE, or MSE) were monitored. Significant changes in performance during such perturbation may indicate unstable performances.

3. Results and Discussion

3.1 Model Training/Generalisation Assessment

The CatBoost model, for the 2000 dataset, demonstrated the strongest predictive performance for estimating As concentrations ($R^2 \approx 0.70$; Table 1), outperforming both the random forest ($R^2 \approx 0.67$) and linear regression model ($R^2 \approx 0.31$). CatBoost also presented the lowest testing error metrics (RMSE ≈ 62.9, MAE ≈ 33.4). For the 2023 dataset, the CatBoost again achieved the highest testing performance ($R^2 \approx 0.79$), although the random forest yielded slightly lower testing error metrics (RMSE ≈ 45.1, MAE ≈ 21.3). Overall, the machine learning models outperformed the linear regression model in terms of predictive performances and error metrics. This reinforces the importance and appropriateness of using machine learning for identifying and ranking different hydrochemical and hydrological controls influencing As concentrations over statistical models. These findings are expected given the ability of machine learning to handle non-linear interactions, as they rely on complex data patterns rather than on linear assumptions.

CatBoost also indicated strong generalisation capabilities for both datasets, indicating stable cross-validation performances ($R^2 \approx 0.60$, 0.75, respectively). Coupled with the high testing R^2, we can confidently conclude high generalisation performance for both CatBoost models. The random forest indicated high generalisation capabilities, however, seeing the relatively lower testing performances, we proceed using a CatBoost. The statistical linear model indicated significantly poor generalisation performances, indicating its vulnerability to overfitting and unstable performances. For information around the specific configurations and hyperparameter tuning metrics for each model, see Tables 2-5.

For our hyperparameter tuning search, we show the optimal CatBoost architecture for this particular predictive task (Table 2). We show a shallow tree depth (≈ 4), moderate learning rate (0.01-0.02), with a relatively large number of boosting iterations (> 500) yields the optimal predictive performances. We show the most appropriate booster type to be "Bernoulli" rather than the conventional "Bayesian" approach. Bernoulli induces more randomness into the tree architecture, forcing decision trees to learn more direct intricate patterns and noise. We show our As predictor being highly skewed (SD = 57.46 µg/L), non-linear, while containing lots of noise. A booster type like Bernoulli can help this CatBoost learn the intricacies of this noise, improving its adaptability to overfitting, hence we see better generalisation performance.

Table SI1. CatBoost, random forest, and linear regression generalisation performances for the 2000 and 2023 datasets comparing sample to feature ratio (SRF), testing, and training metrics.

Model	Year	Target	Features	SRF	$R^2 \approx$ (Test)	$R^2 \approx$ (k-fold)	RMSE ≈ (Test)	MAE ≈ (Test)
CatBoost	2000	As	GWL, NH_4, Fe, Mn	115	0.70	0.60 ± 0.05	62.9	33.4
Random Forest	2000	As	GWL, NH_4, Fe, Mn	115	0.67	0.60 ± 0.05	65.4	35.4
Linear Regression	2000	As	GWL, NH_4, Fe, Mn	115	0.31	0.18 ± 0.25	70.5	34.5
CatBoost	2023	As	GWL, NH_4, Fe, Mn	86	0.79	0.75 ± 0.05	60.4	26.3
Random Forest	2023	As	GWL, NH_4, Fe, Mn	86	0.71	0.70 ± 0.05	45.1	21.3
Linear Regression	2023	As	GWL, NH_4, Fe, Mn	86	0.40	0.30 ± 0.10	528	95.5

Table SI2. CatBoost configuration outlining the splitting ratios and hyperparameter specifications.

Model	Year	Target	Train:Test Ratio	Learning Rate (%)	Iterations (Trees)	l2_leaf_reg	Max_depth	Sub-sample	Booster Type	Random Seed
CatBoost	2000	As	85%:15%	0.012	1200	4	4	0.75	Bernoulli	88
CatBoost	2023	As	90%:10%	0.020	500	10	4	0.70	Bernoulli	5

Table SI3. Random forest configuration outlining the splitting ratios and hyperparameter specifications.

Model	Year	Target	Train : Test Ratio	n_ estimators	max_ depth	min_ samples_split	min_samples_ leaf
Random Forest	2000	As	85% : 15%	500	None	2	1
Random Forest	2023	As	80% : 20%	100	None	2	1

Table SI4. Linear regression configuration outlining the splitting ratios and hyperparameter specifications.

Model	Year	Target	Train : Test Ratio	fit_ intercept	copy_x	Random_ State	n_feautures
Linear Regression	2000	As	85% : 15%	True	True	88	4
Linear Regression	2023	As	80% : 20%	True	True	95	4

3.2 Overfitting Assessment

3.2.1. Learning curves

Previously, we examined the ability of our CatBoost models to generalise to unseen data and across cross-validation folds, with both models showing strong generalisation (Table SI1). The purpose of this exercise was to assess their susceptibility to overfitting. Given the relatively small training dataset sizes for the 2000 (n = 461) and 2023 (n = 344) CatBoost models, it's important to evaluate overfitting risk using learning curves (Figure SI2). In these curves, we compared the testing and training performances with increasing sample sizes (e.g., 50, 100, 150, 200); ideally, training performance should remain slightly higher than testing, and neither curve should fluctuate sharply with increasing sample sizes.

These learning curves can help identify whether the CatBoost tends to overfit when trained on limited data. For example, if the gap between training and testing significantly narrows around a sample size of approximately 300, this suggests training a CatBoost on fewer than 300 rows can increase the likelihood of overfitting. The learning curves for both 2000 and 2023 CatBoost models indicate very mild to almost no overfitting (Figure SI2). First, the difference between the training and testing R^2 is minimal ($R^2 \pm 0.5$), showing that both Catboost models are learning data patterns that generalise rather than fitting noise. Second, the slightly higher training performance compared with testing performance further supports the argument the models are not overfitting and memorising the training data. Finally, both training and validation curves exhibit small, smooth fluctuations, with no erratic deviations, indicating both models behave consistently as the number of training samples increase. We show both models benefit with more training data; however, performance does plateau (approx. $R^2 \approx 0.70$, $R^2 \approx 0.80$, respectively). Such plateauing may be caused by several factors. First, the plateau may reflect the information limit of the current number of features used. Second, this plateauing may reflect the upper performance bound achievable with tree-based models like CatBoost. Lastly, the intrinsic noise in the target variable for As makes predicting As levels challenging, meaning such noise may cap the ability of continuously improving such machine learning models.

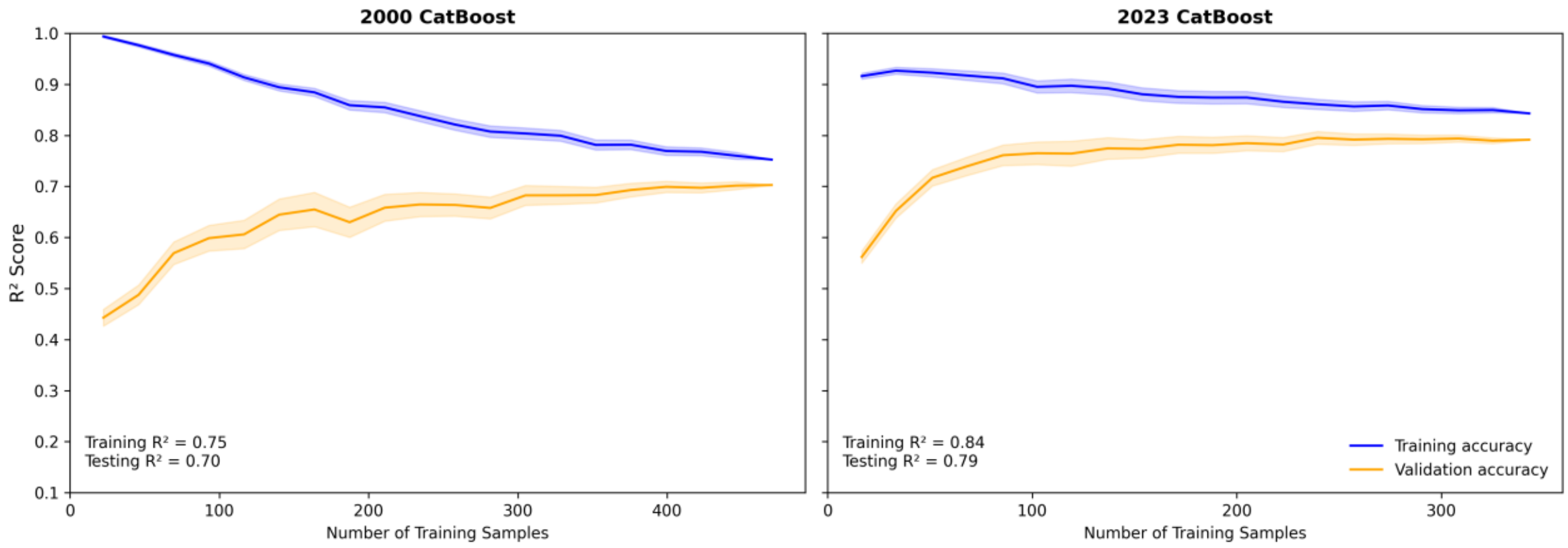

Figure SI2. Learning curves comparing training (blue) and validation (yellow) performance for the CatBoost models applied to the 2000 and 2023 datasets, using iteratively increasing training sample sizes (i.e., 100, 200, 300, 400). The target variable is As, with GWL, Fe, Mn, and NH_4 used as predictors. Shaded regions around the training and validation fitted lines represent the variability (confidence bands) across the k-fold cross-validation splits.

3.2.2. Permutation Testing

We also examined whether our two CatBoost models overfitted the training dataset by conducting a permutation test, where we randomly shuffled all target As variables, while keeping the predictor variables fixed. Essentially, we shuffled the As values and randomly assigned them to a row of predictor variables. Ideally, after such shuffling, the CatBoost performance should immediately drop, as this shuffling means there is no meaningful pattern or relationships between the shuffled As variables and its assigned combination of features. If such performance does not drop, this can indicate whether the CatBoost is memorising noise and possibly overfitting.

After shuffling such target inputs, we observe a considerable decline in the testing R^2 performance (Figure SI3). Specifically, the mean CV R^2 for the 2000 and 2023 CatBoost models was approximately $R^2 \approx -0.15$ and $R^2 \approx -0.10$, respectively (Figure SI3). These findings conclude that the previous CatBoost models, trained on the correct target to feature pairs, were learning real and meaningful relationships. When these pairs were removed through random shuffling, these relationships diminish, with model performance collapsing as expected. This confirms such models were not genuinely overfitting caused by memorising random target noise, and their original predictive performance is far above what would occur by chance.

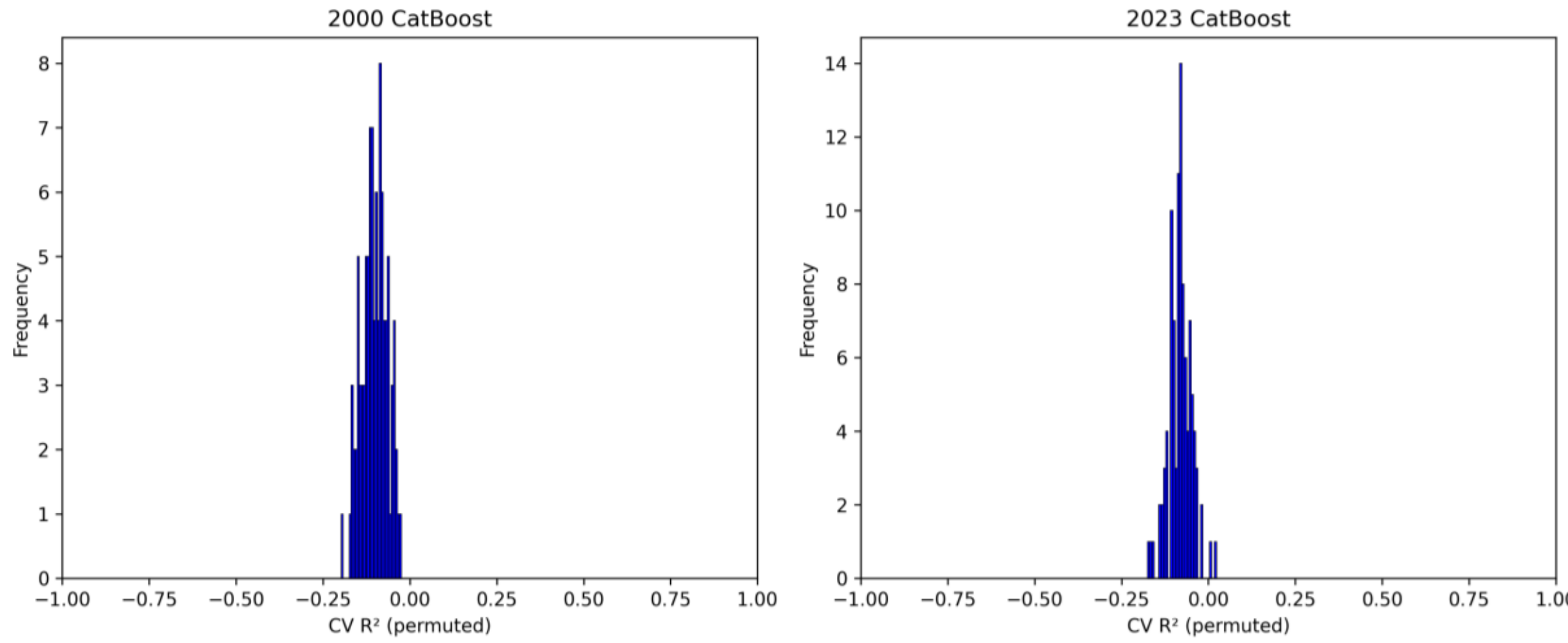

Figure SI3. CV R^2 score for the CatBoost trained on the 2000 and 2023 dataset computed after random shuffling. We shuffled 100 times, so the frequency of each bar equates to 100 total shuffles.

3.3 Model Robustness Assessment

The SFR, defined as the number of data rows per predictor, is a useful indicator of CatBoost robustness across different dataset sizes. Higher SFR values (i.e., > 10) typically produce more stable model behaviour and reduced variance by allowing the CatBoost to learn more data patterns and less noise. To examine this effect, we trained CatBoost models (using identical configurations; Table 2), on the 2000 and 2023 datasets, while systematically increasing the SFR and evaluating how training and testing performance change. For the 2000 dataset, we compared SFR of 20, 40, 60, 80 100, including the maximum attainable SFR of 117 used in our final model. For the 2023 dataset, we evaluated SFR of 20, 40, 60, 80 for our 2023 dataset, alongside the maximum SFR of 85 used in the final CatBoost 2023 model.

For the 2000 CatBoost model, increasing the SFR from 20 to 100 gradually improved testing performance ($R^2 \approx 0.54 \rightarrow 0.68$) while reducing training performance ($R^2 \approx 0.96 \rightarrow 0.77$; Figure SI4). This convergence between training and testing indicates reduced overfitting likelihood and improvement in model robustness, as performance gap decreases with increasing SFR. Reviewing the performance gap between training and testing suggests SFR values below ~ 60 are prone to overfitting, whereas SRF ≥ 60 yields significantly more stable and robust performances.

For the 2023 CatBoost model, testing performance remains relatively stable across SFR thresholds; however, increasing the SFR still reduces the convergence performance gap between training and testing, particularly when SFR > 60. These findings reveal several conclusions. First, model robustness is strongly influenced by SFR, with lower SFR, causing high divergences between training and testing performances. Second, although a SFR of 20 is considered acceptable in the general guidance of Zhu et al., (2023), our results show for this particular research task, such a ratio is insufficient for generating accurate and reliable predictions. Lastly, our analysis shows that SFR above ~60 are necessary to achieve CatBoost robustness between testing and training performances for both datasets.

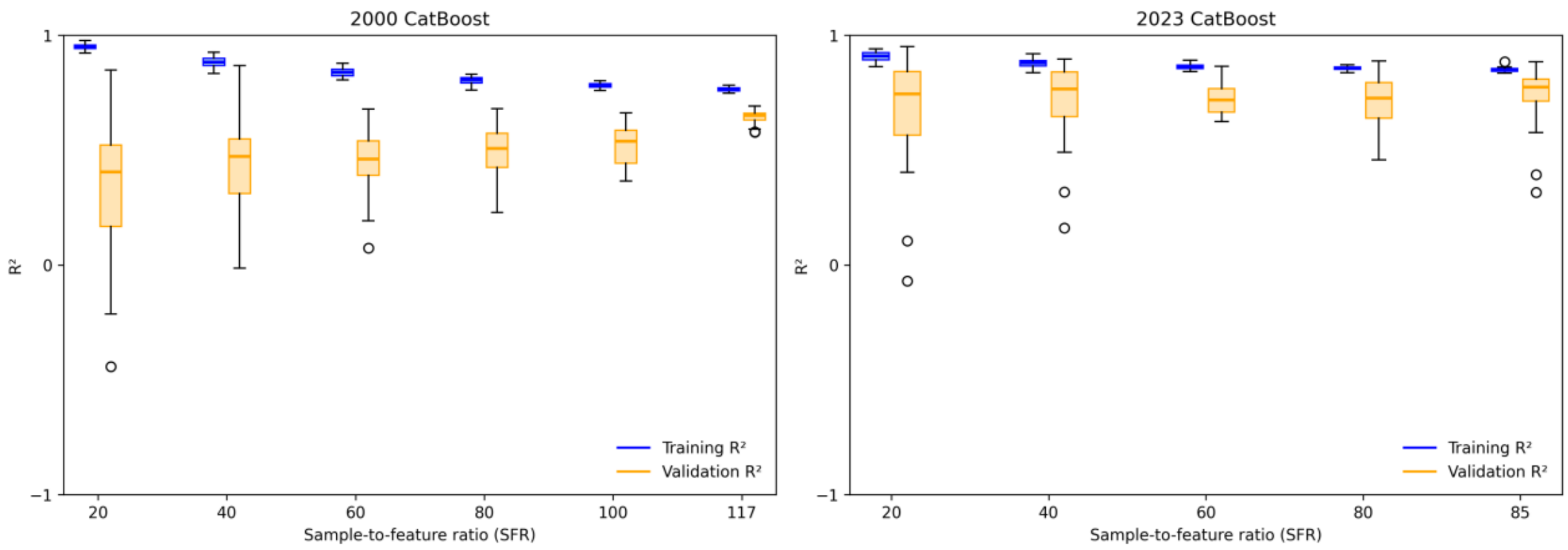

Figure SI4. Incrementally increasing CatBoost SFR, cross-comparing training (blue) and validation (yellow) performance scores, for both 2000 and 2023 datasets.

3.4. Model Stability Assessment

(i) Regularisation Robustness

We examined the sensitivity of both CatBoost models to different regularisation strengths (Table SI5). Regularisation was varied to deliberately adjust model complexity: stronger regularisation penalties can penalise large or overly flexible model behaviour, essentially forcing the model to become simpler. A stable CatBoost model should maintain consistent performance scores, irrespective of varying regularisation penalties. Based on our previous generalisation (Section 3.1) and overfitting tests (3.2), we expect stable performances, irrespective of adjusting for regularisation strength. We applied L2 regularisation, controlled by the hyperparameter *l2_leaf_reg*, which is highly effective at regularising boosted tree models containing few input features. Regularisation strength was varied from very weak (3) to strong (50), and for each setting we evaluated CatBoost performance (R^2) and error metrics (RMSE, MAE) for both datasets (Table SI5).

CatBoost models showed consistent performances across different regularisation strengths. For the 2000 CatBoost model, predictive performance deviated slightly from very weak to very strong regularisation strength (R^2 = 0.71 → 0.70, respectively), while maintaining consistent error metrics (RMSE = 62.92 → 64.67; MAE = 33.28 → 34.55; Table SI5). For the 2023 CatBoost model, predictive performance did not deviate between very weak and very strong regularisation strengths (R^2 = 0.79 → 0.79), while error metrics remained relatively stable (RMSE = 59.24 → 67.43; MAE = 25.52 → 29.76). These findings conclude both CatBoost models are not dependent on low regularisation or high model complexity to achieve stable performances.

Table SI5. CatBoost stability performance under varying L2 regularisation penalties for the 2000 and 2023 datasets.

	CatBoost 2000			CatBoost 2023		
Regularisation Penalty	R^2 (Test)	RMSE (Test)	MAE (Test)	R^2 (Test)	RMSE (Test)	MAE (Test)
Very Weak: l2_leaf_reg = 3	0.71	62.92	33.28	0.79	59.24	25.52
Weak: l2_leaf_reg = 5	0.70	62.93	33.36	0.79	58.33	25.01
Moderate: l2_leaf_reg = 10	0.71	62.82	33.46	0.79	60.38	26.29
Strong: l2_leaf_reg = 20	0.71	63.50	33.88	0.78	62.74	27.64
Very Strong: l2_leaf_reg = 50	0.70	64.67	34.55	0.79	67.43	29.76

(ii) Input Sensitivity Robustness

Our final stability assessment involved perturbing the input features by ± 2%, examining how sensitive the CatBoost models were to perturbations of the input features. Hydrochemical and hydrological data inherently contain noise and variation due to analytical uncertainty, sampling variability, and instrument precision, while also varying extensively within the environment. For instance, groundwater hydrochemical properties may drastically change across small spatial scales, making it challenging to interpolate such data. A robust CatBoost model should therefore maintain similar predictive performance when exposed to such input perturbations. To test this, all the input features were simultaneously perturbed by ±2%, and the resulting predictive accuracy (R^2) and error metrics (RMSE, MAE) were evaluated. If the CatBoost is stable and not overly sensitive to noise, predictive performance should not deviate despite such perturbations.

Irrespective of slight input perturbations, both CatBoost models demonstrated stabilised performances (Table SI6). For the 2000 CatBoost, predictive performance deviated slightly ± 2% perturbation (R^2 = 0.70 → 0.71), with consistent error performances (RMSE = 63.5 → 63.3; MAE = 33.8 → 33.4; Table SI6). Similarly, the 2023 CatBoost had no performance deviations (R^2 = 0.79 → 0.79), with small variations in error metrics (RMSE = 62 → 59.5; MAE = 27.1 → 25.7). Such results conclude that these CatBoost models are noise-stable, capable of generalising reliability.

Table SI6. CatBoost performance evaluation under ± 2% perturbation scenarios for the 2000 and 2023 datasets.

	CatBoost 2000			CatBoost 2023		
Perturbation	R^2 (Test)	RMSE (Test)	MAE (Test)	R^2 (Test)	RMSE (Test)	MAE (Test)
- 2%	0.70	63.5	33.8	0.79	62	27.1
0 %	0.70	62.9	33.4	0.79	60.4	26.3
+ 4%	0.71	63.3	33.4	0.79	59.5	25.7

Conclusion

Our analysis evaluated (i) generalisation, (ii) overfitting, (iii) robustness, (iv) and stability of the two CatBoost models used for variable-importance analysis in the 2000 and 2023 datasets. The machine learning models (CatBoost and random forest) consistently outperformed the linear regression model, achieving higher predictive performances and lower error metrics. This confirms that using traditional statistical models are not appropriate for variable importance ranking using these data types, where such target-to-feature associations are typically non-linear. Both CatBoost models indicated strong generalisation performances, mild overfitting, and stable, robust predictive performance. Overall, the models are well suited for deriving variable importance rankings, but our results recommend sample-to-feature ratios above 60 to ensure reliable and accurate performances.
