# Supplementary material for: Modeled Groundwater Decline is Associated with Lower Arsenic Concentrations in Hanoi
Source: Environ Sci Technol. 2026 Oct 18;60(43):30887–900. doi: 10.1021/acs.est.6c07700 (PMC13738782; doi:10.1021/acs.est.6c07700)
Supplement: Supplementary file 2 [file es6c07700_si_003.pdf]

Supplementary Material for

Modeled Groundwater Decline is Associated with Lower Arsenic Concentrations in Hanoi

David Haaf[1,2], Michael McGlade*[1,2], Athena A. Nghiem[1,2,3], Duyen Thi Vu[4], Tobias Steinert[1,2], Viet Hung Pham[4], Linh Khanh Bui[5], Thanh Ngoc Tong[6], Duong Du Bui[5], Andrea Stenke[1,2], Michael Berg[1], Lenny H. E. Winkel*[1,2]

[1] Eawag: Swiss Federal Institute of Aquatic Science and Technology, Department of Water Resources and Drinking Water, 8600 Dubendorf, Switzerland.

[2] Institute of Biogeochemistry and Pollutant Dynamics, Department of Environmental Systems Science (D-USYS), ETH Zurich, 8092 Zurich, Switzerland.

[3] Department of Geoscience, University of Wisconsin-Madison, Madison, WI 53706, USA.

[4] Key Laboratory of Analytical Technology for Environmental Quality and Food Safety Control (KLATEFOS), VNU University of Science, Hanoi 11417, Vietnam.

[5] Water Research Institute, National Center for Water Resources Planning and Investigation (NAWAPI), Hanoi 10000, Vietnam.

[6] Vietnam Water and Environment Association, Hanoi 11307, Vietnam.

* Corresponding authors: mmcglade@ethz.ch, Lenny.Winkel@eawag.ch

List of Tables

Table SI1. Overview of the two hydrochemical datasets sampled in 2000 and 2023 for Greater Hanoi used in this study as input for interpretable machine learning.

Study	Number of Wells	Sampling Period	Hydrochemical Parameters	Measurement Technique
Tong et al. 2001 (NHEGD)	470	Nov. 2000	As[a], Fe, Mn	AAS
			NH_4^+, HCO_3^-	Field Laboratory
Our Study (2023 Dataset)	434	Feb. - June 2023	Eh, EC, pH, DO, HCO_3^-	Field probes, Testkits
			Cl^-, Br^-, SO_4^{2-}, NO_3^--N	IC
			As[b], Fe, Mn, Na, K, Ca, Mg	ICP-MS
			DOC	TOC analyzer
			NH_4^+	Photometer
			CH_4	GC-2000

[a] Limit of Quantification: 1 µg L^{-1}; [b] Limit of Quantification: 0.8 µg L^{-1}

Table SI2. Calibration performance and instrument detection limits for analytes measured in the Red River Delta 2023 sampling campaign.

Element	Calibration R^2	Instrument Detection Limit (µg/L)
As	0.999	0.01
B	0.999	0.09
Br	0.999	0.55
Ca	0.999	5.20
Fe	0.999	0.05
K	0.999	1.83
Li	0.999	0.02
Mg	0.999	0.21
Mn	0.999	0.03
Na	0.999	0.28
P	0.998	0.16
Si	0.999	5.92
Sr	0.999	0.08
S	0.999	0.22

Table SI3. Descriptive statistics of all measured hydrochemical parameters in the field with multi-prob sensor and lab for the 2023 Red River Delta campaign. Min values with a < indicate detection limits (SD = standard deviation).

Parameter	Unit	n	Mean	SD	Median	Min	Max
pH	-	434	7.0	0.6	7.0	4.0	8.9
Eh	mV	434	8.0	102	-24	-195	296
DO	mg L^{-1}	434	1.2	1.2	0.6	0.2	7.0
EC	S cm^{-1}	434	565	323	536	47	2119
Temperature	°C	433	26	1.0	26	11	30
Alkalinity (HCO_3^-)	mg L^{-1}	432	327	209	305	3.0	1592
F^-	mg L^{-1}	434	0.2	0.2	0.2	< 0.05	1.8
Cl^-	mg L^{-1}	434	28	26	21	< 0.5	174
Br^-	mg L^{-1}	434	0.1	0.1	0.1	< 0.05	1.1
NO_3^-	mg L^{-1}	434	0.8	2.6	0.1	< 0.1	24
SO4	mg L^{-1}	434	11	19	1.6	< 1.0	121
DOC	mg L^{-1}	434	2.4	3.6	1.2	< 0.5	38
Li	µg L^{-1}	434	4.0	4.0	5.0	< 1.0	59
B	µg L^{-1}	432	15	13	11	< 5.0	136
Na	mg L^{-1}	434	25	16	21	1.9	93
Mg	mg L^{-1}	434	16	9.7	15	0.4	58
Al	µg L^{-1}	434	27	24	15	< 15	124
Si	mg L^{-1}	434	15	5.0	15	3.3	30
Cl	mg L^{-1}	434	27	24	20	< 2.0	169
K	mg L^{-1}	434	3.4	3.3	2.5	0.3	34.1
Ca	mg L^{-1}	434	40	33	32	0.8	160
P	mg L^{-1}	434	0.5	0.7	0.2	< 0.01	6
S	mg L^{-1}	434	3.1	5.5	0.4	< 0.02	33
V	µg L^{-1}	434	0.3	0.6	0.1	< 0.1	8
Cr	µg L^{-1}	434	0.6	1.5	< 0.4	0.1	18
Mn	mg L^{-1}	434	0.7	1.0	0.4	< 0.01	9.7
Fe	mg L^{-1}	434	7.3	8.5	5.2	< 0.01	62
Cu	µg L^{-1}	434	0.3	0.3	0.2	< 0.1	5
Zn	µg L^{-1}	434	4.0	11	2.0	< 2.0	142
As	µg L^{-1}	434	41	74	8.0	< 0.8	440
Br	mg L^{-1}	434	0.1	0.1	0.1	< 0.03	1
Sr	µg L^{-1}	434	0.2	0.1	0.2	0.01	0.8
Se	µg L^{-1}	434	0.4	0.6	0.5	< 0.5	12
Mo	µg L^{-1}	434	1.3	2.4	0.4	< 0.03	16
Cd	µg L^{-1}	434	0.2	0.1	0.2	< 0.05	1.7
Ba	mg L^{-1}	434	0.2	0.2	0.2	< 0.1	1.9
Pb	µg L^{-1}	434	0.1	0.2	0.1	< 0.1	2
U	µg L^{-1}	434	0.3	1.0	0.01	<0.01	11
NH_4^+	mg L^{-1}	434	7.5	16	1.2	< 0.2	163
CH_4	mg L^{-1}	427	5.3	10	0.13	< 0.13	49

Table SI4. Descriptive statistics of all measured hydrochemical parameters in the field with multi-prob sensor and lab for the 2000 Red River Delta campaign. Min values with a < indicate detection limits (SD = standard deviation).

Parameter	Unit	n	Mean	SD	Median	Min	Max
pH	–	470	6.14	0.73	6.3	3.7	8.3
As	µg L^{-1}	470	40	57	14	1.0	331
EC	µS cm^{-1}	470	189	167	140	0	1078
Fe	mg L^{-1}	470	11	19	6.5	0	244
Mn	mg L^{-1}	470	0.84	1.1	0.46	0.01	9.4
Alkalinity (HCO_3^-)	mg L^{-1}	470	186	133	173	-30	754
Eh	mV	470	32	31	26	-88	151
NH_4^+	mg L^{-1}	470	7.9	26	0.65	0	260
DO	mg L^{-1}	470	1.90	1.4	1.6	0	8.4

List of Figures

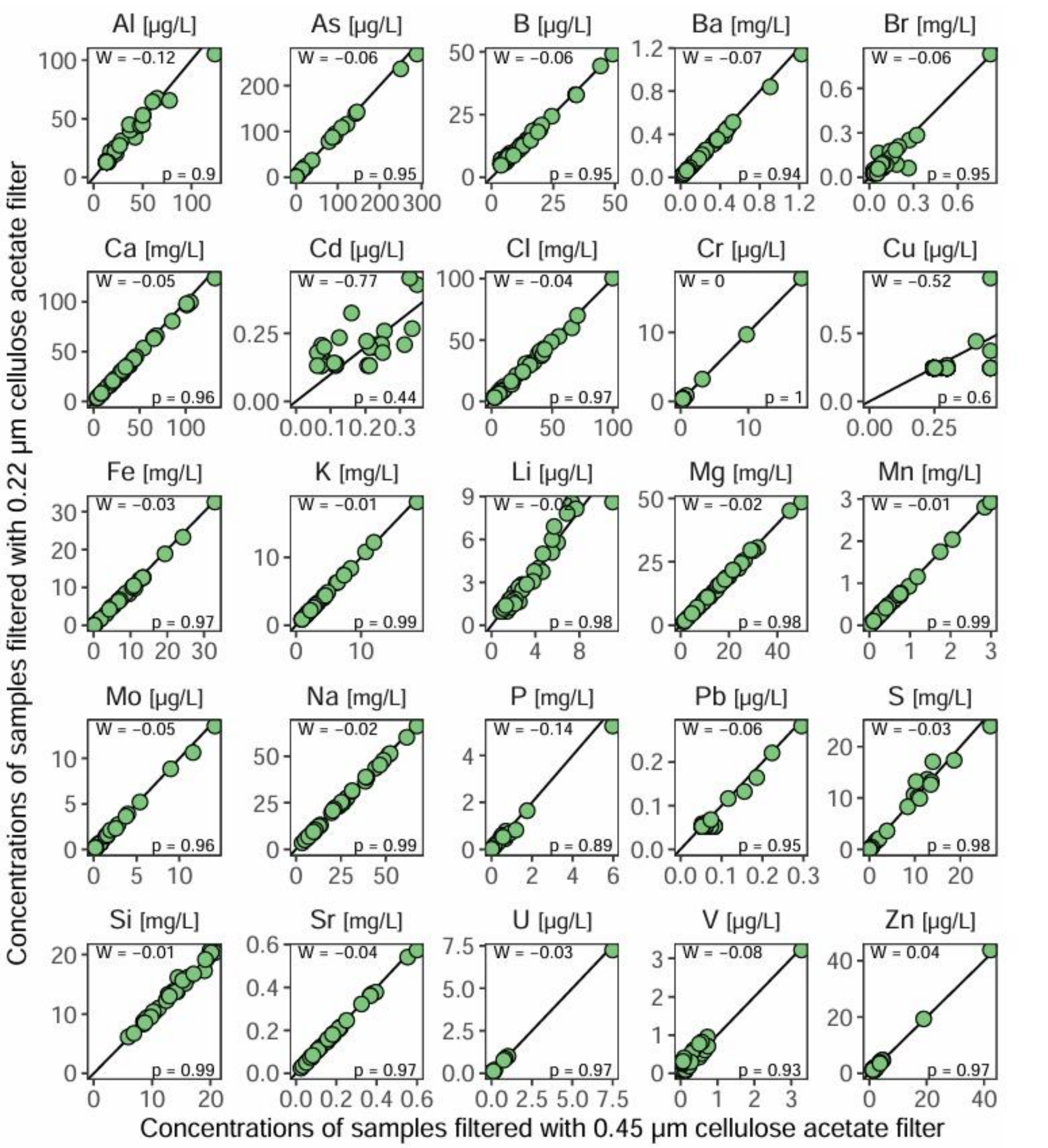

Figure SI1. Comparison of measurements by filter sizes (0.22 mm and 0.45 mm) for elements measured with ICP-MS/MS. The text represents the result of the Wald statistics and the respective p-value of the Wald test (Wald, 1943). The black line represents the 1:1 line.

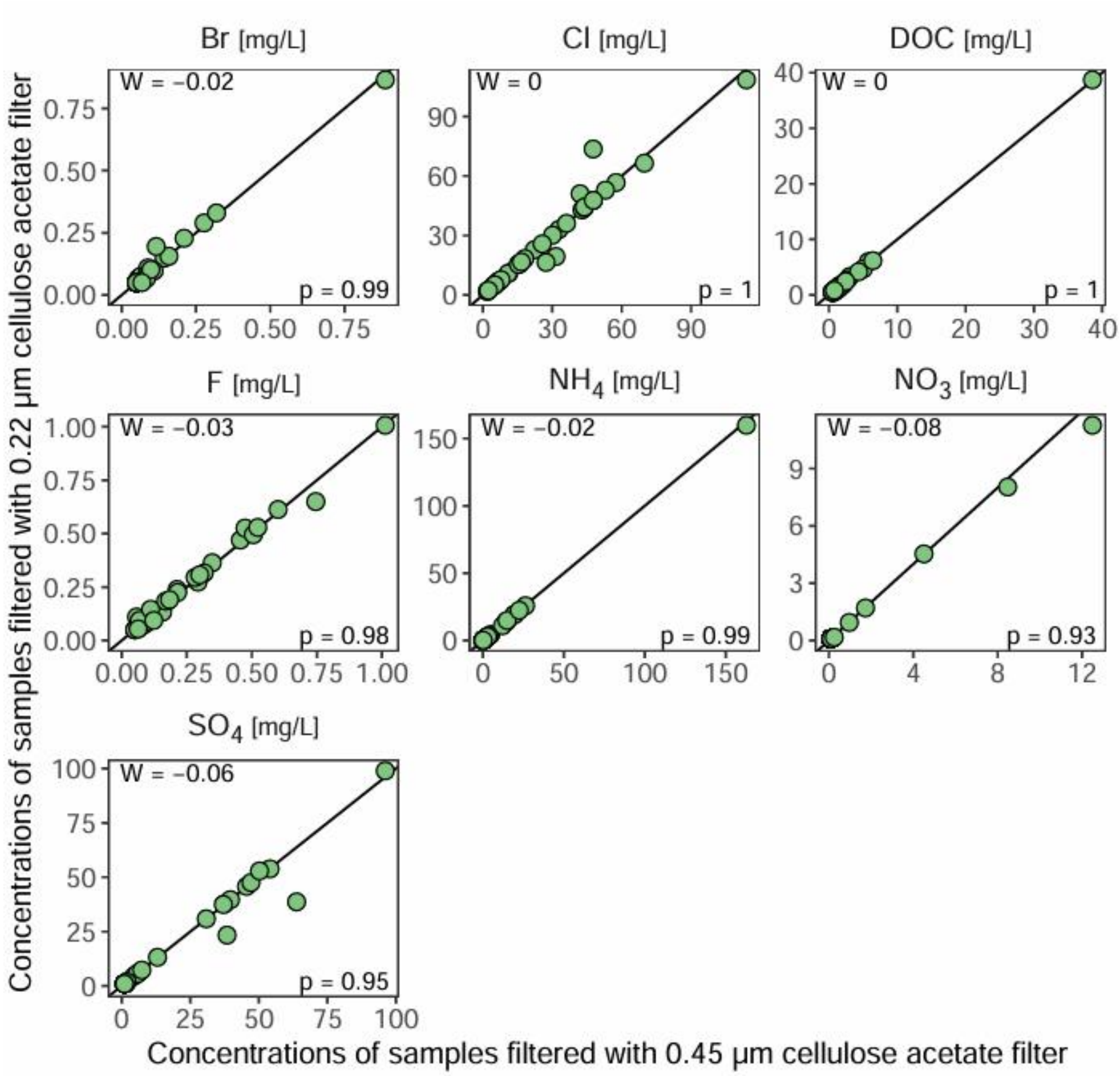

Figure SI2. Comparison of measurements by filter sizes (0.22 m and 0.45 m) for elements measured with IC (Cl, Br, F, NO3, SO4), TOC (DOC) and photometry (NH4). The text represents the result of the Wald statistics and the respective p-value of the Wald test. The black line represents the 1:1 line.

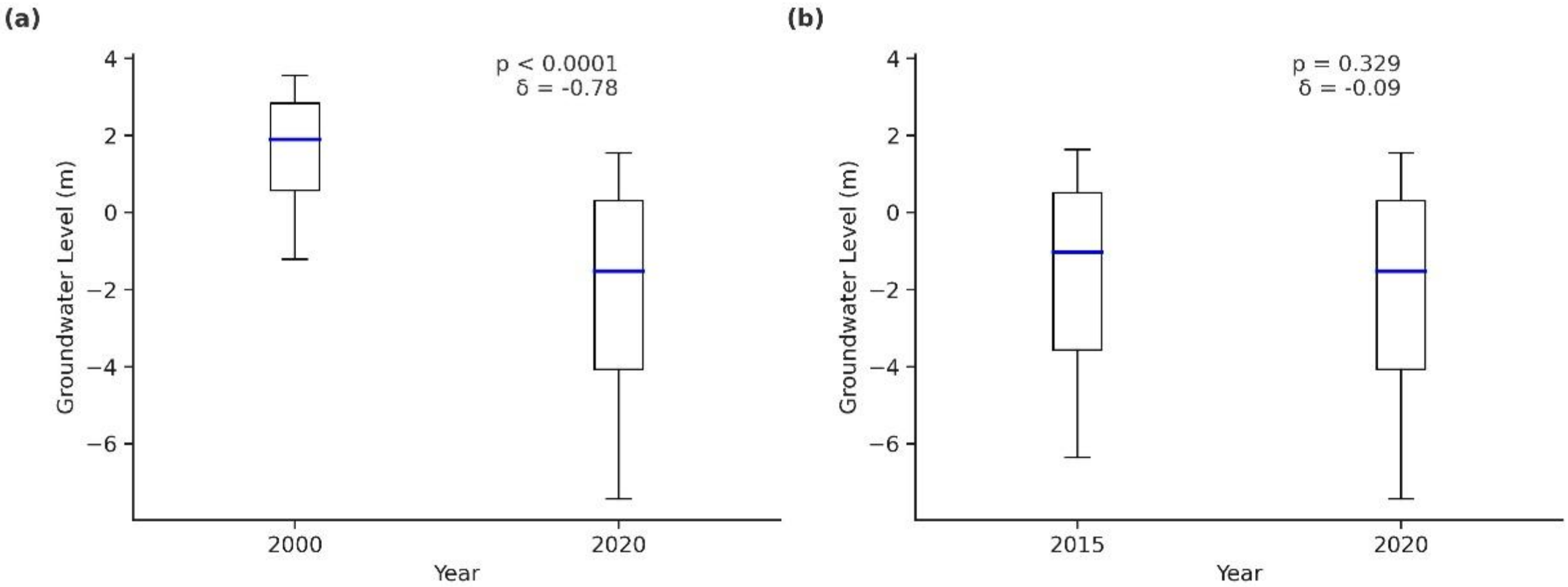

Figure SI3. (a) Median MODFLOW-simulated groundwater levels (GWL) at the grid cells corresponding to the 434 wells sampled in 2023, comparing 2000 and 2020 GWL, showing significant long-term decline. (b) compares such GWL changes between 2015 and 2020, indicating a stabilising decline, with no statistically significant changes.

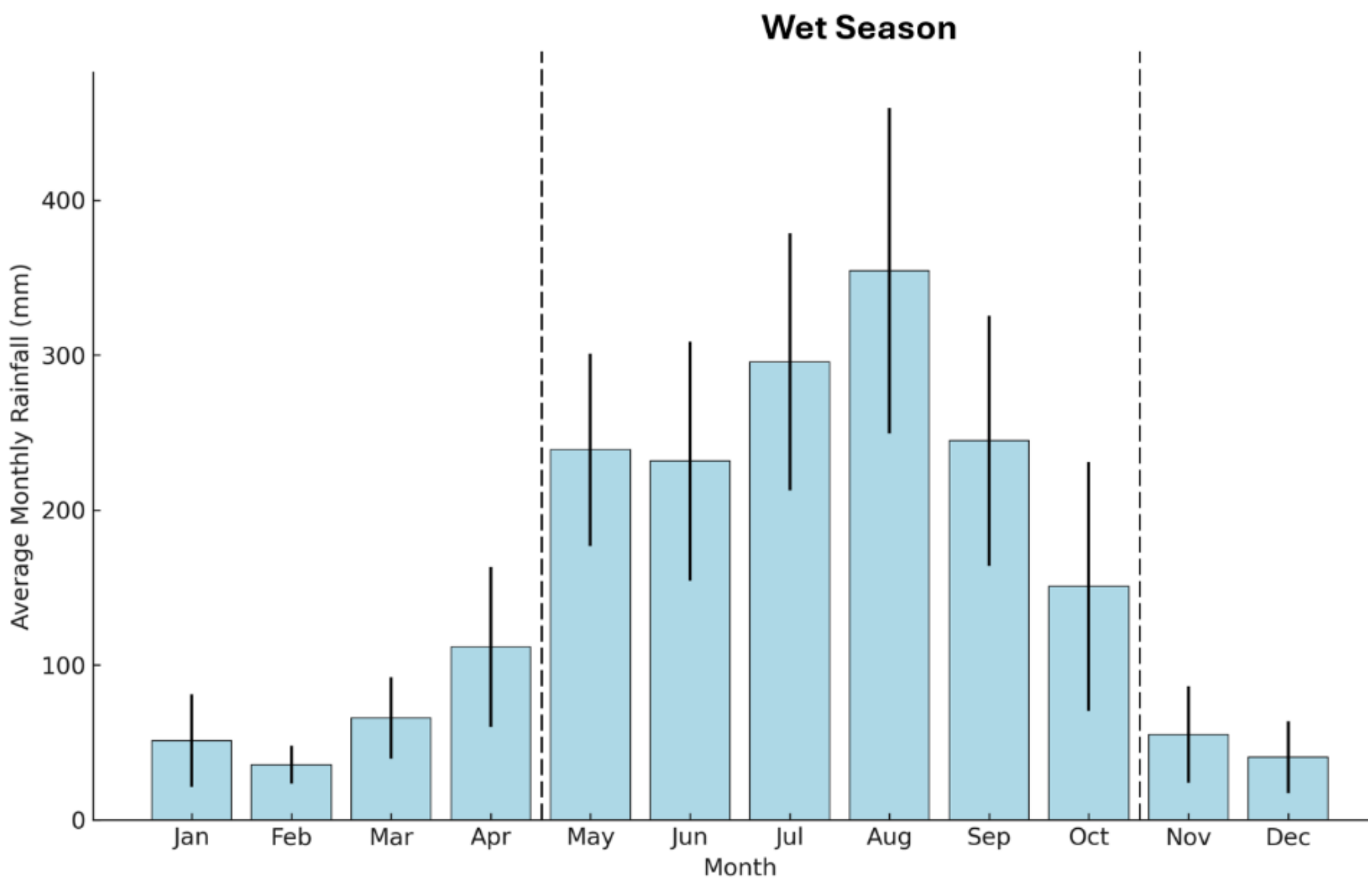

Figure SI4. Mean monthly rainfall for the central Hanoi region, with standard deviation bars, indicating the wet season (May–October) and dry season (November–April). The mean rainfall values were obtained from daily rainfall recordings from ECMWF between 1990-2023.

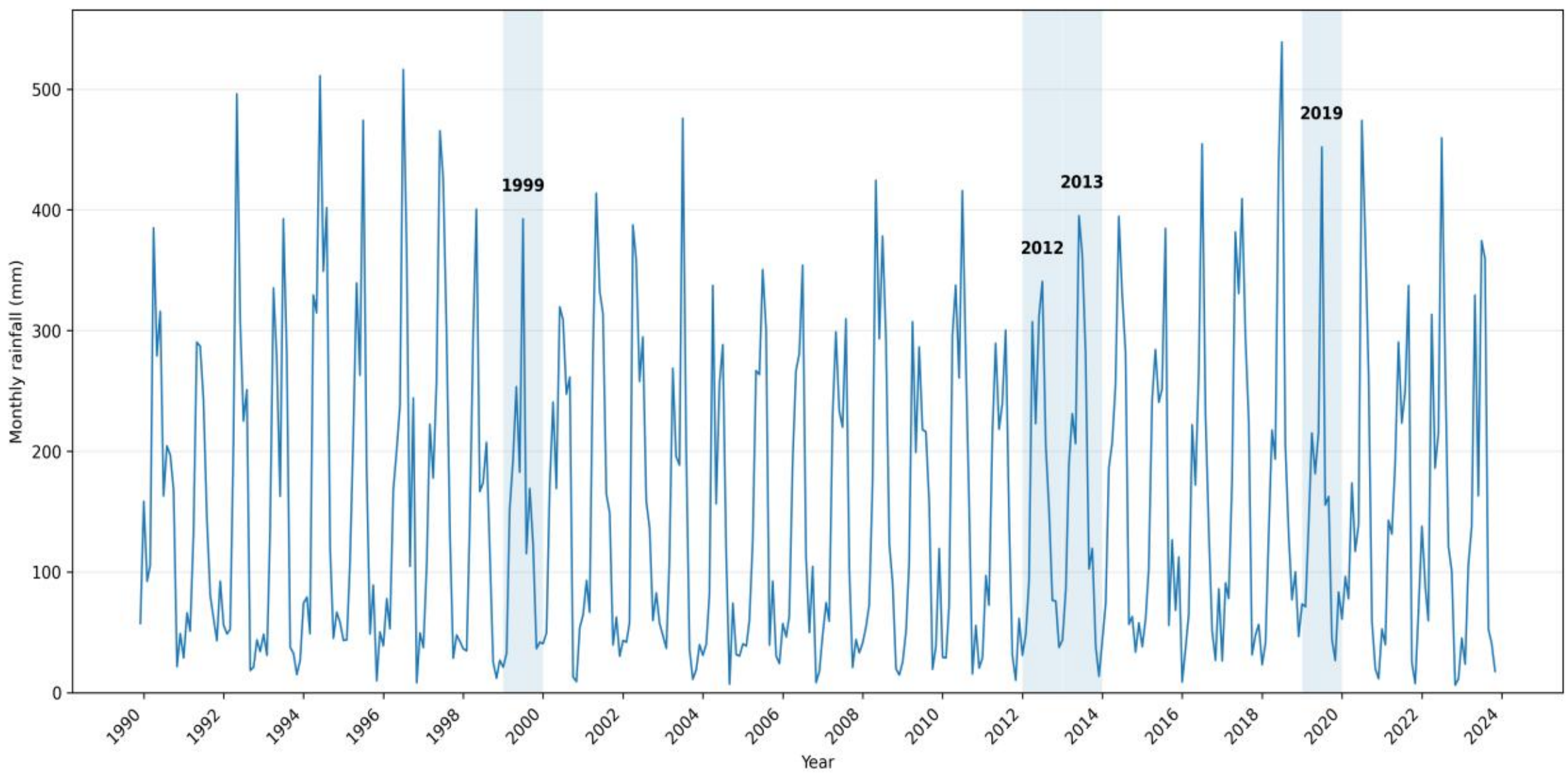

Figure SI5. Monthly ERA5 rainfall across the Hanoi study area (20.75–21.50° N; 105.75–106.50° E) from 1990 to 2023. Shaded periods indicate the antecedent years examined for potential unusually wet or dry conditions preceding the 2000, 2013/2014, and 2020 groundwater-level periods.[49]

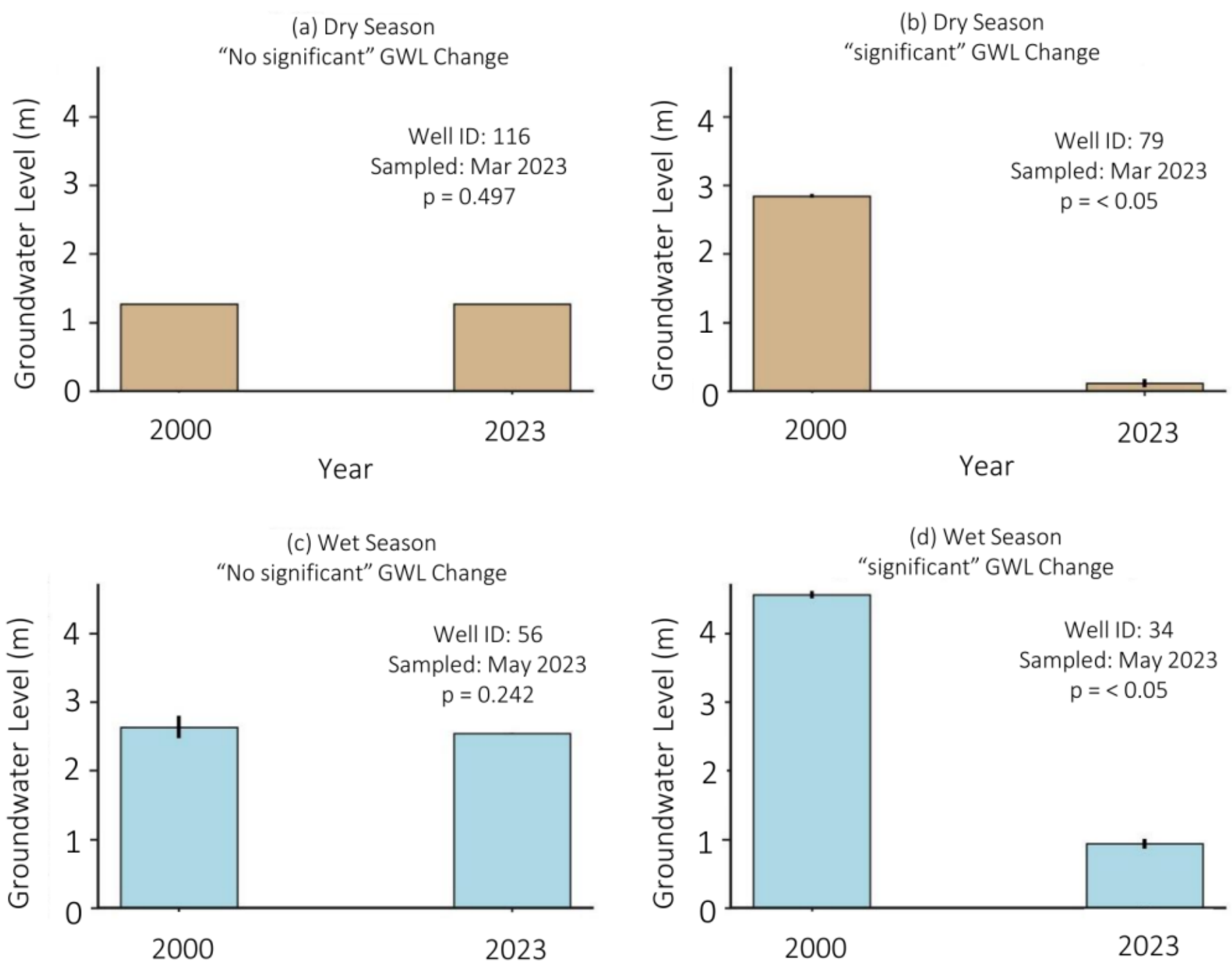

Figure SI6. Examples of wells sampled in the 2023 campaign grouped into the four groundwater-level change categories. (a) Well ID 116, sampled in March 2023 (dry season), showed "no significant" change in groundwater levels between 2000 and 2020. (b) Well ID 79, sampled in March 2023 (dry season), showed a "significant" groundwater level change. (c) Well ID 56, sampled in May 2023 (wet season), showed "no significant" change. (d) Well ID 34, sampled May 2023 (wet season), showed "significant" change.

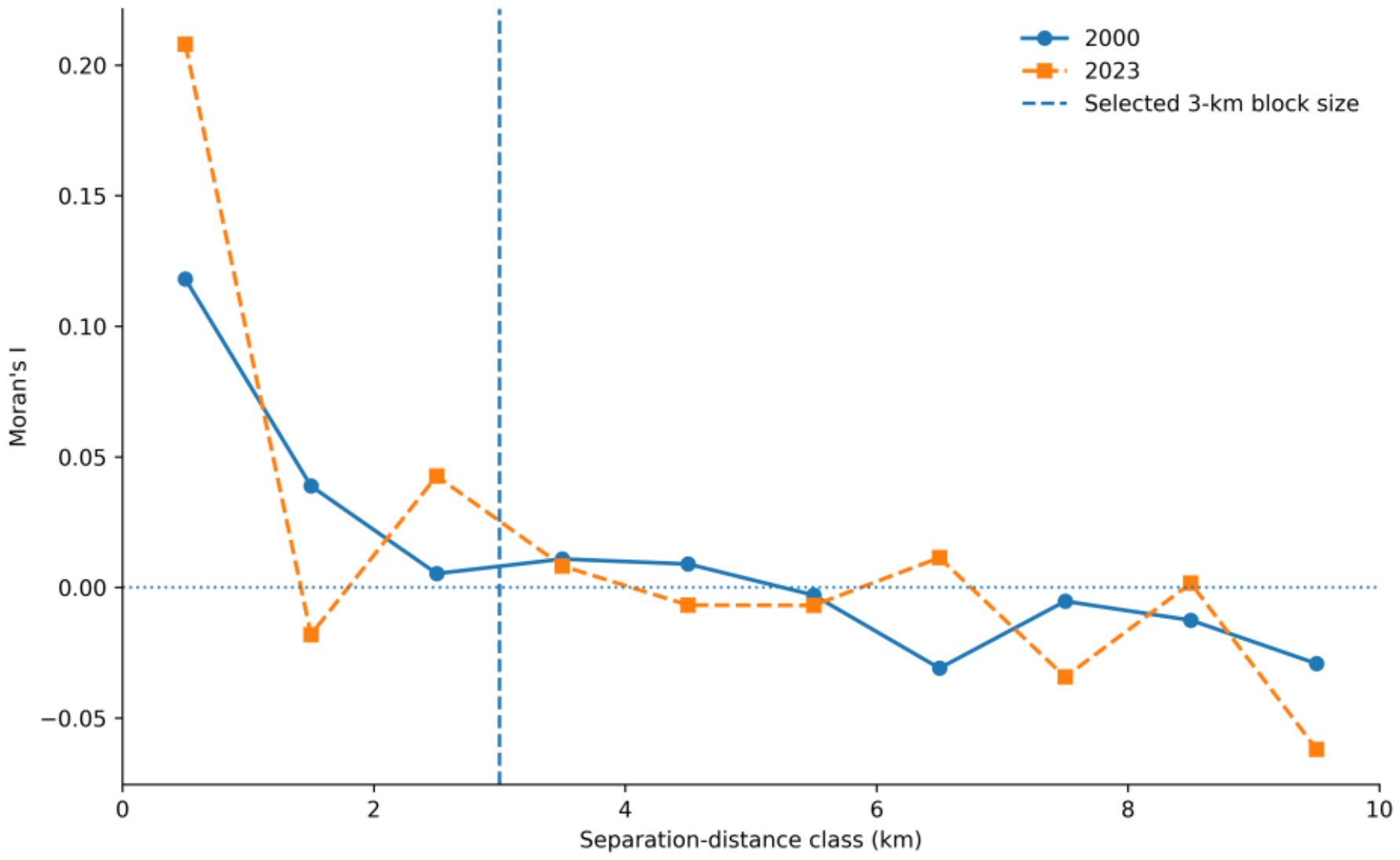

Figure SI7. Distance-class Moran's I analysis of groundwater As concentrations in the 2000 and 2023 campaigns, used to support selection of the 3 km spatial block size.

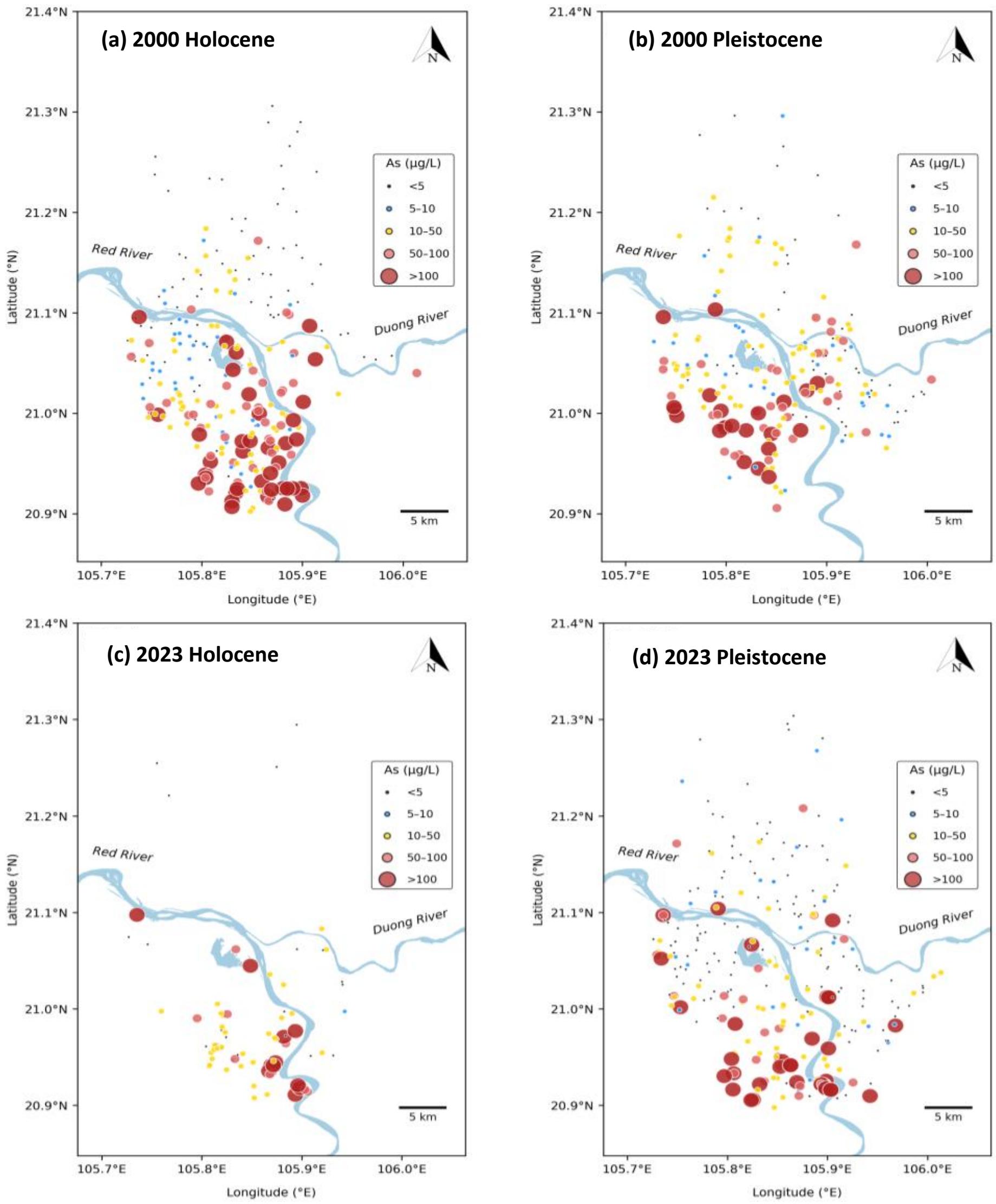

Figure SI8. Arsenic concentrations in wells sampled in (a) 2000 Holocene, (b) 2000 Pleistocene, (c) 2023 Holocene, and (d) 2023 Pleistocene.

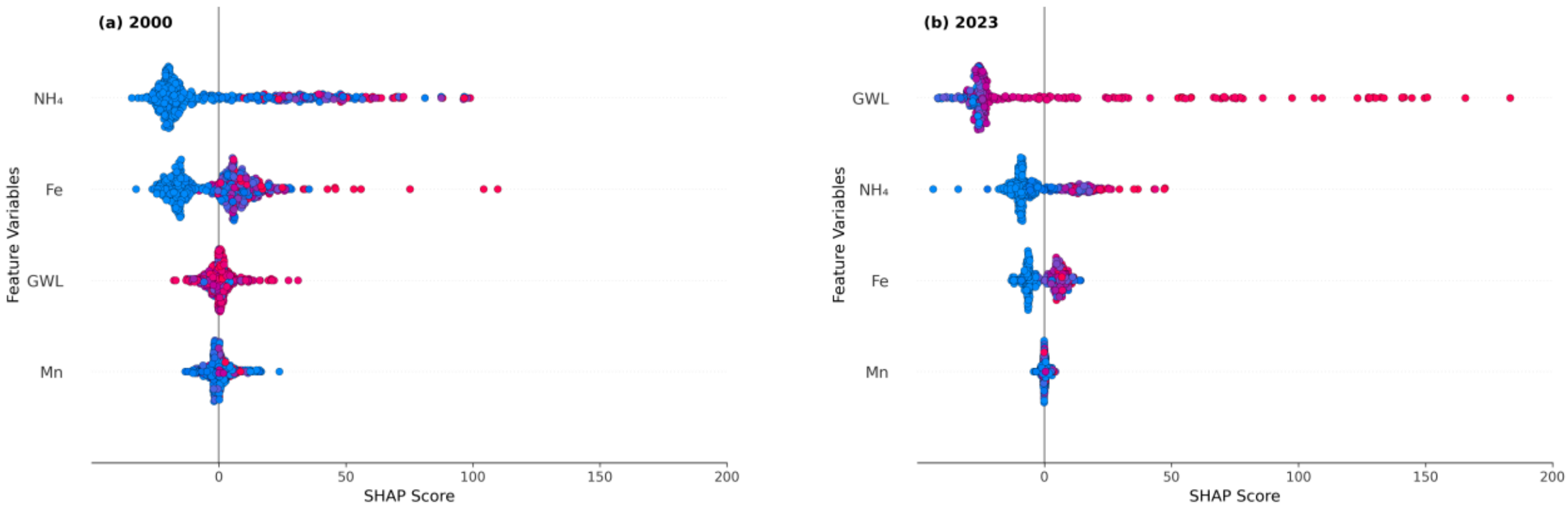

Figure SI9. Random forest SHAP analysis of the 2000 (a) and 2023 (b) datasets, illustrating the relative importance of NH_4, Fe, GWL, and Mn in relation to predicted As concentrations.

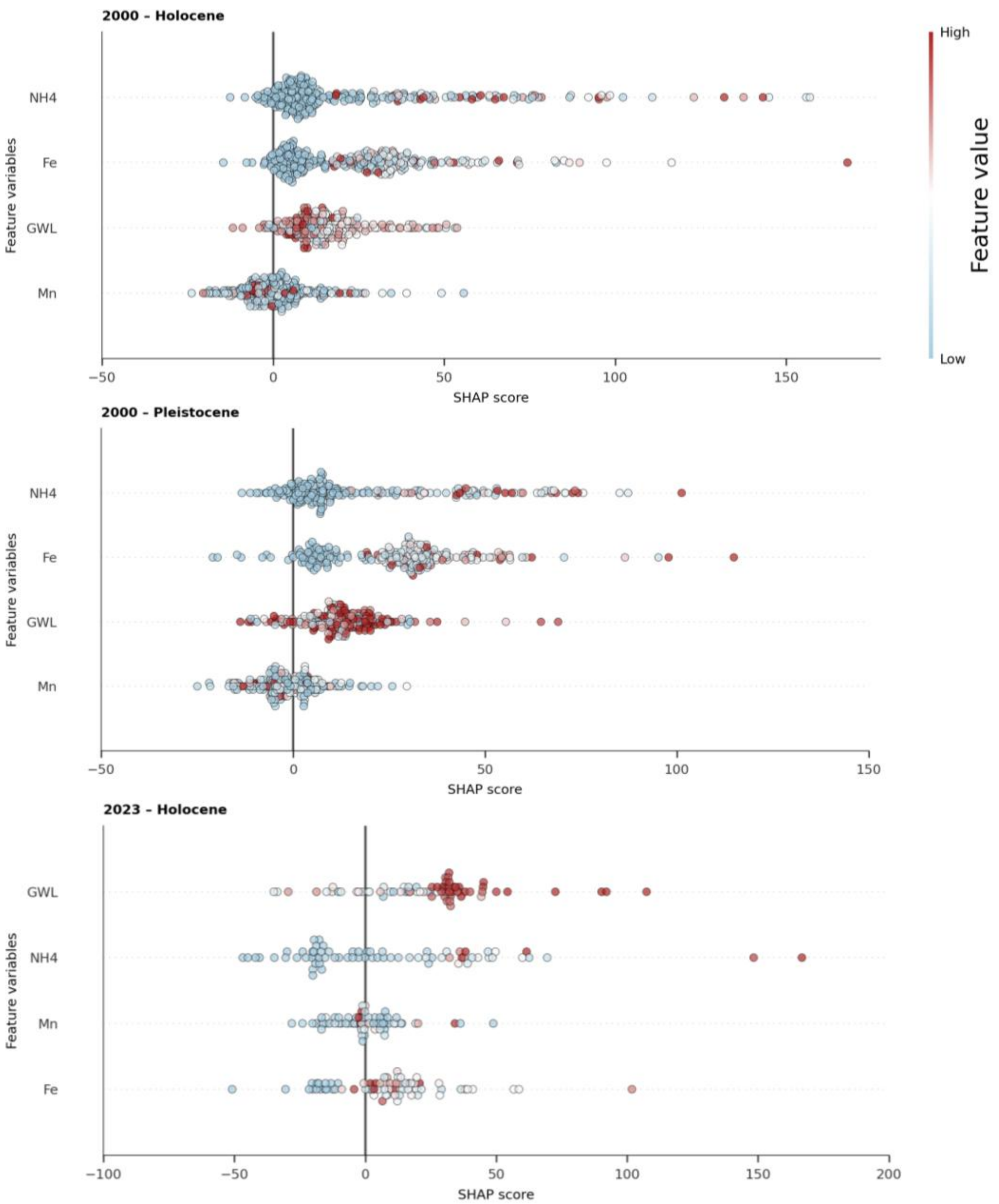

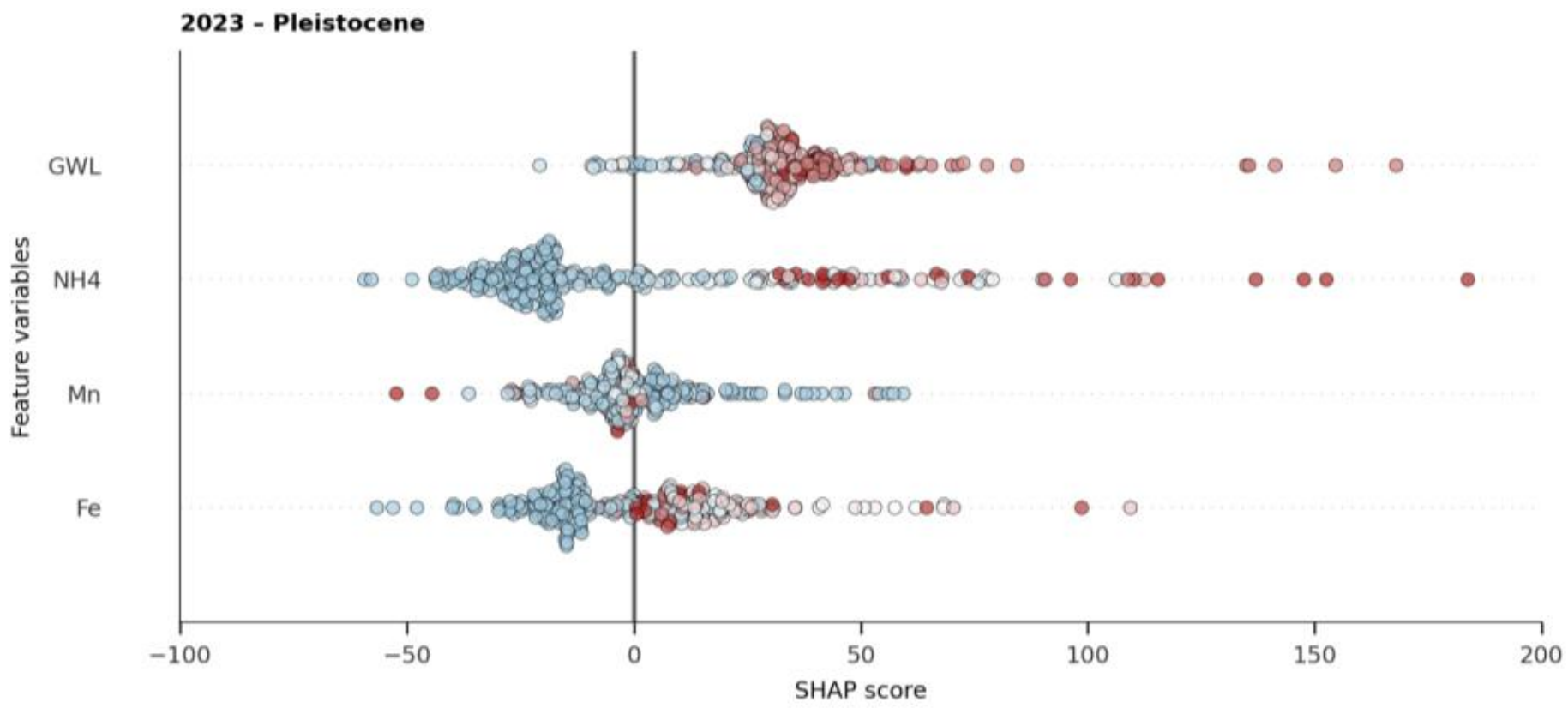

Figure SI10. Mean SHAP scores ranking the importance of groundwater level (GWL), NH_4, Fe, and Mn in influencing As concentrations for wells tapping into Holocene and Pleistocene aquifers in 2000 and 2023. NH_4, Fe, and Mn are expressed as input concentrations, while GWL represents estimated changes in groundwater depth (m below ground). Blue dots indicate low values of each feature (i.e., GWL, NH_4, Mn, and Fe), while red dots indicate high values. Positive SHAP scores indicate that the feature increases As concentrations, while negative scores indicate a decreasing effect

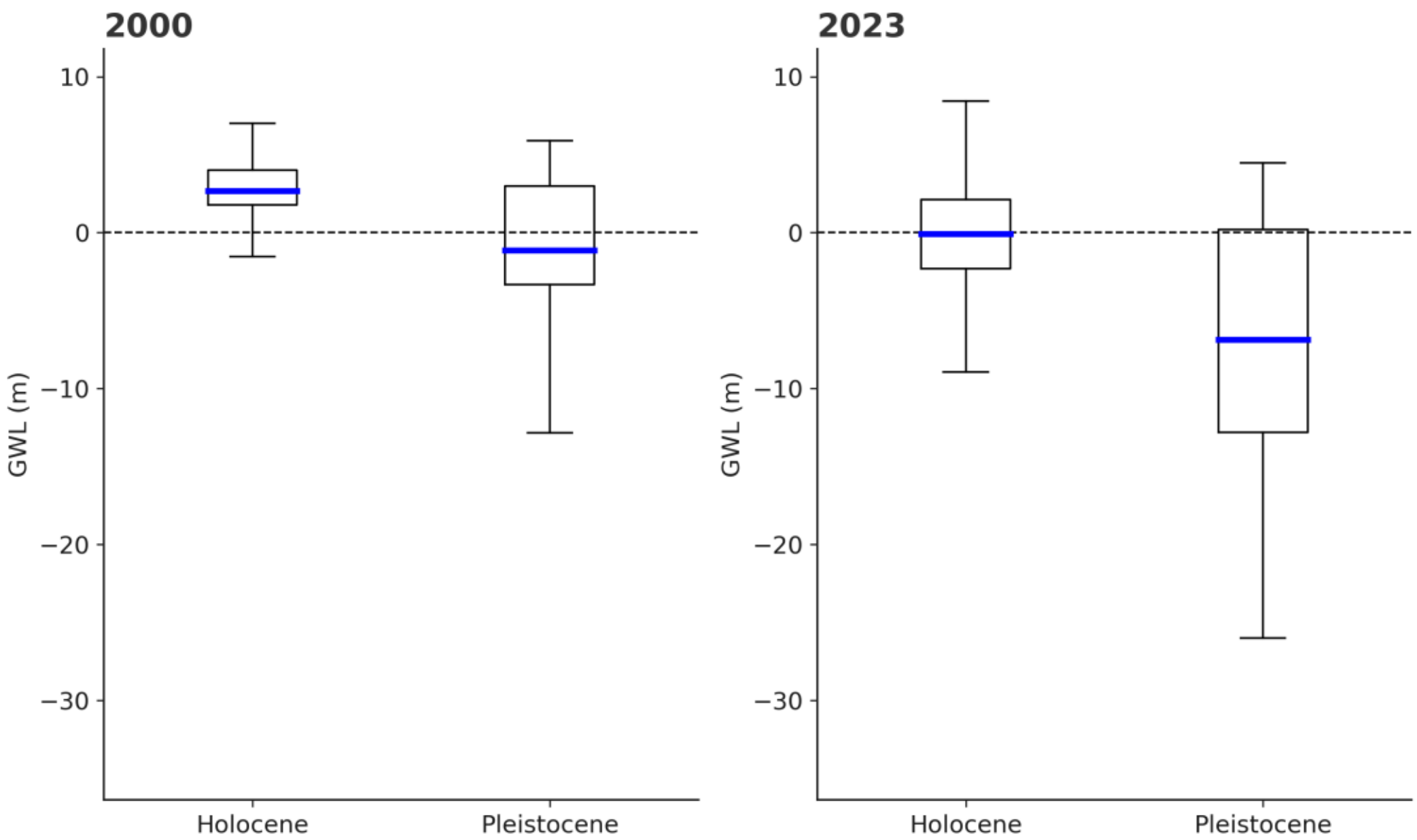

Figure SI11. Average groundwater levels (GWL; blue line) for all wells sampled in 2000 and 2023 in the Holocene and Pleistocene aquifers. GWL values above 0 m indicate water levels standing above sea level (asl)

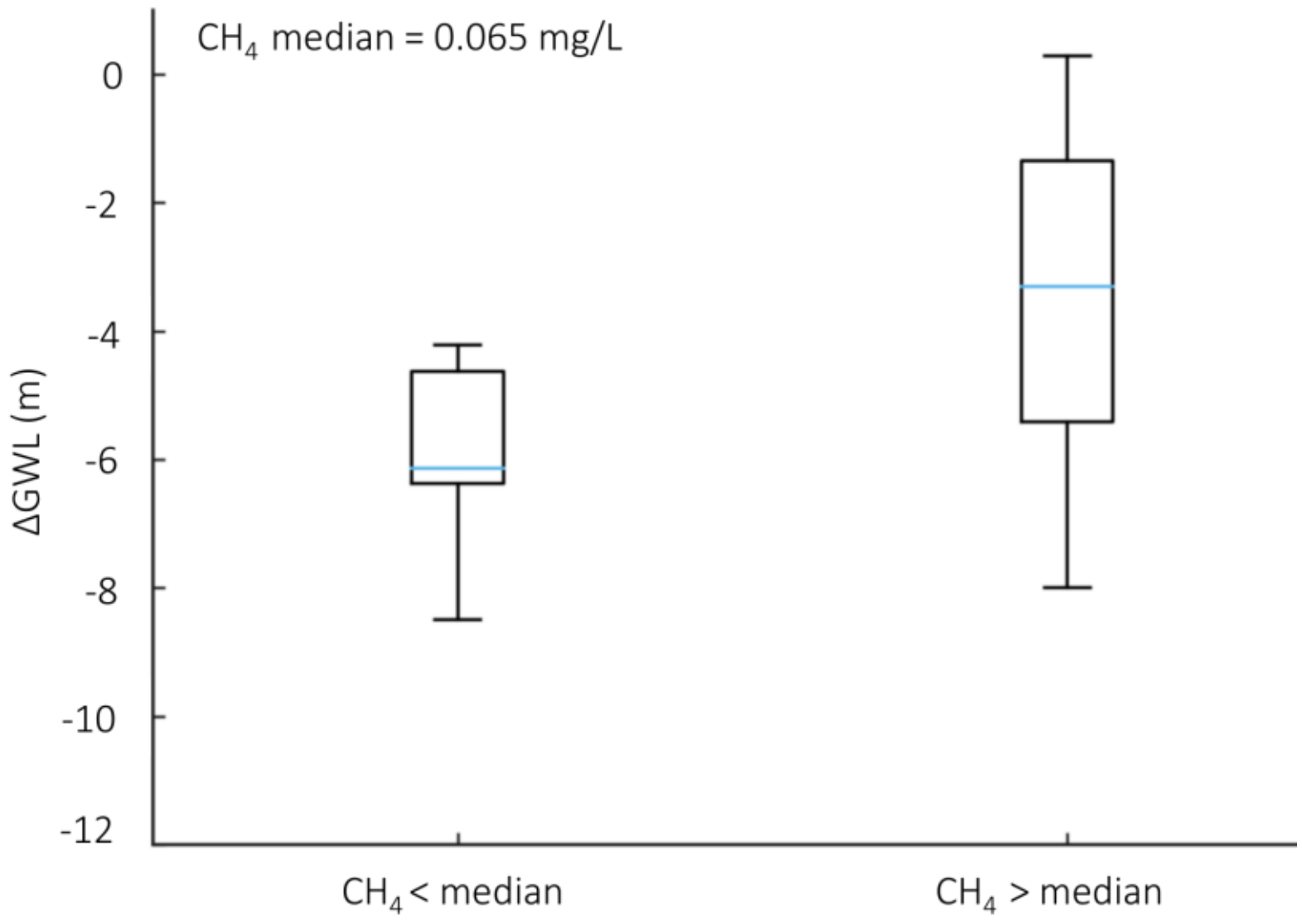

Figure SI12. Comparing GWL changes (m) above and below median CH_4 (0.065mg/L) concentrations. The difference between both thresholds was statistically significant ($t = 2.612$, $p = 0.01$). Number of wells in the "CH_4 < median" class is 128, number of wells in the "CH_4 > median" class is 119.

Supplementary Methods

2.2. Groundwater Sampling Procedure

Groundwater sampling followed established protocols conducted for the Red River Delta.[24,44,47] Wells were pumped until field parameters stabilized and were measured using a portable meter Hach HQ40d, including pH (PHC201), Eh (MTC301), dissolved oxygen (LDO101), electrical conductivity (CDC401), and built-in temperature sensor. Alkalinity (HCO_3) was determined using a Merck MColortest kit. Groundwater was collected in a bucket pre-rinsed with well water, and samples were drawn into pre-cleaned 60 mL syringes. For all wells, six aliquots were filtered with pre-washed disposable 0.45 µm cellulose acetate filters (BGB Analytik). For 25 wells in the village of Van Phuc, samples were additionally filtered using 0.22 µm cellulose acetate filters to assess potential filtration effects on measured concentrations. No systematic differences were observed between 0.45 µm and 0.22 µm filtrations; results were therefore considered comparable (Figures SI1&SI2, Supplementary Material).

For each well, four aliquots were analysed at Eawag (Switzerland), and two at KLATEFOS, VNU University of Science (Vietnam). At Eawag, the first aliquot, collected in 15 mL Nalgene polypropylene bottles, was used for trace element and cation analysis and acidified to pH < 2 with 1 % v/v HNO_3. A second aliquot for dissolved organic carbon was collected in 60 mL Nalgene polypropylene bottles and acidified to pH < 2 with 1 % v/v HCl. A third aliquot, also collected in 60 mL Nalgene polypropylene bottles and not acidified, was used for anion analysis. The fourth aliquot was collected for stable water isotope analysis in 4 mL amber glass vials and was not acidified. At the sampling sites, aliquots were collected for NH_4^+ and CH_4 analyses. The NH_4^+ aliquot was collected in 20 mL HDPE bottles (Wheaton) and acidified to pH < 2 with 1 % v/v H_2SO_4 (95 % Merck puriss, p.a.). Methane samples were collected directly from the sampling tube connected to the well by inserting a needle into 5.9 mL evacuated glass vials (Labco 819W), filled to two-thirds volume and leaving one-third as headspace.

2.3. Laboratory Measurements

Trace element and major cations (i.e., S, P, Li, B, Na, Mg, Al, Si, K, Ca, V, Cr, Mn, Fe, Cu, Zn, As, Br, Sr, Se, Mo, Cd, Ba, Pb, U) were determined by inductively coupled plasma mass spectrometry (ICP-MS; Agilent 8900 Triple Quadrupole), equipped with an automated in-line dilution system (prepFast, dilution factor = 10; limits of quantification). Major Anions (i.e., Cl^-, Br^-, F^-, SO_4^{2-}, and NO_3^-–N) were measured by ion chromatography (Metrohm 930 Compact IC Flex). All batches used the same calibration, internal standards, and quality control set-ups. Multi-element calibration standards (Bernd-Kraft) were used for quantification, with Sc, Rh, and Lu as internal standards to monitor signal stability in ICP-MS analyses. Quality control involved using certified reference materials (i.e., NIST 1643f, Sigma-Aldrich Multielement Ion Chromatography Anion Standard, and As reference samples ARS 29-32) analyzed every 25 samples per batch. Certified single-element reference materials were used for quality control for Si and U. Dissolved organic carbon (DOC) was measured by catalytic combustion with infrared CO_2 detection using a total C and N analyzer (Shimadzu TOC-L CSH). Ammonium was measured by spectrophotometry (Shimadzu UV-1800) and CH_4 by GC-2014 (Shimadzu) with a Porapak T column following the headspace equilibrium method.[48] At least three quality-control checks were performed within each of the four analytical batches. Calibration curves showed $R^2 \geq 0.998$, and recoveries exceeded 95% for all measured elements across all batches. Concentrations below the analyte-specific LOQ were assigned the corresponding LOQ value. Field and laboratory blanks showed no anomalies.

2.4. Antecedent Rainfall Analysis

Long-term rainfall patterns (1990–2023) show a consistent wet season between May and October and a dry season between November and April (Figure SI4, Supplementary Material). These periods were therefore used as fixed seasonal windows for comparing GWL change in this study. Similar seasonal classifications have been reported in previous studies conducted in Greater Hanoi.[50] To assess whether antecedent rainfall could influence GWL during 2000, 2013/14, and 2020, monthly ERA5 precipitation data (1990–2023) were examined across Greater Hanoi (20.9–21.5° N; 105.8–106.3° E).[49] Rainfall in preceding years (1999, 2012, 2013, and 2019) was compared with long-term climatology using Wilcoxon signed-rank tests, pairing each month with the corresponding climatological month. May–October monsoonal rainfall was assessed similarly. No significant differences were observed for antecedent years or monsoonal periods (all $p > 0.05$; Figure SI5), indicating no evidence of exceptionally wet or dry antecedent conditions. Nevertheless, antecedent climatic variability remains a source of uncertainty when interpreting GWL differences between study periods.

2.5. Double/Debiased Machine Learning Workflow

In the DML analysis, groundwater As concentration was specified as the outcome variable, with NH_4, Fe, Mn and GWL considered in turn as the treatment variable and the remaining three predictors included as controls. The DML analysis was conducted separately using the full analysis dataset for each sampling campaign and independently of the train/test splits used to evaluate CatBoost performance. DML was implemented using five-fold cross-fitting. For each fold, CatBoost nuisance models were trained using four folds of the data and used to generate predictions for the remaining held-out fold. This was repeated across all five folds, with each fold serving once as the held-out fold for prediction. We also reported the DML estimates with two-sided 95% confidence intervals and p-values, calculated from the corresponding DML standard errors using Wald inference.

Supplementary Results

Section 3.1

To assess aquifer classification (Holocene or Pleistocene), we fitted sensitivity models including aquifer as a fifth categorical predictor. In five-fold cross-validation, adding aquifer increased mean R^2 by 0.037 in 2000 and 0.003 in 2023. Predictions from the four- and five-predictor models were strongly correlated (Spearman's $\rho = 0.977$ and 0.988 for 2000 and 2023, respectively). Aquifer ranked third in 2000, behind NH_4 and Fe, and fifth in 2023. Thus, aquifer provided modest predictive information in 2000 but little in 2023, with limited influence on performance and predictions. Lower GWL values were associated with lower predicted As in both aquifers (Figure SI10). This analysis included 258 Holocene and 204 Pleistocene wells in 2000 and 70 Holocene and 274 Pleistocene wells in 2023. Because numerical well depth data were unavailable for 2000 and only aquifer classifications were provided, these results should be interpreted cautiously. Within each campaign, GWL SHAP distributions did not differ significantly between aquifers. Although the Holocene aquifer may be more susceptible to groundwater replacement and flushing under pumping-induced hydraulic gradients, potentially due to greater hydraulic connectivity to surface recharge zones, including rivers and lakes, stratified SHAP results did not establish that GWL–As associations differed between aquifers. Average GWL declines from 2000 to 2023 were broadly comparable in Holocene and Pleistocene aquifers (3.5 and 2.9 m, respectively; Figure SI11).
